# Comparing outcomes and costs among warfarin-sensitive patients versus warfarin-insensitive patients using The Right Drug, Right Dose, Right Time: Using genomic data to individualize treatment (RIGHT) 10K warfarin cohort

**DOI:** 10.1371/journal.pone.0233316

**Published:** 2020-05-19

**Authors:** Kristi M. Swanson, Ye Zhu, Ricardo L. Rojas, Jennifer L. St. Sauver, Suzette J. Bielinski, Debra J. Jacobsen, Sue L. Visscher, Liewei Wang, Richard Weinshilboum, Bijan J. Borah

**Affiliations:** 1 Robert D and Patricia E Kern Center for the Science of Health Care Delivery, Mayo Clinic, Rochester, MN, United States of America; 2 Division of Health Care Policy and Research, Department of Health Sciences Research, Mayo Clinic, Rochester, MN, United States of America; 3 Division of Biomedical Statistics and Informatics, Department of Health Sciences Research, Mayo Clinic, Rochester, MN, United States of America; 4 Division of Epidemiology, Department of Health Sciences Research, Mayo Clinic, Rochester, MN, United States of America; 5 Division of Clinical Pharmacology, Department of Molecular Pharmacology and Experimental Therapeutics, Mayo Clinic, Rochester, MN, United States of America; Universita degli Studi di Roma Tor Vergata, ITALY

## Abstract

Oral anticoagulant (OAC) therapy has been the main treatment approach for stroke prevention for decades. Warfarin is the most widely prescribed OAC in the United States, but is difficult to manage due to variability in dose requirements across individuals. Pharmacogenomics may mitigate risk concerns related to warfarin use by fostering the opportunity to facilitate individualized medicine approaches to warfarin treatment (e.g., genome-guided dosing). While various economic evaluations exist examining the cost-effectiveness of pharmacogenomics testing for warfarin, few observational studies exist to support these studies, with even fewer using genotype as the main exposure of interest. We examined a cohort of individuals initiating warfarin therapy between 2004 and 2017 and examined bleeding and cost outcomes for the year following initiation using Mayo Clinic’s billing and administrative data, as well the Mayo Clinic Rochester Cost Data Warehouse. Analyses included descriptive summaries, comparison of characteristics across exposure groups, reporting of crude outcomes, and multivariate analyses. We included N = 1,143 patients for analyses. Just over a third of our study population (34.9%) carried a warfarin-sensitive phenotype. Sensitive individuals differed in their baseline characteristics by being of older age and having a higher number of comorbid conditions; myocardial infarction, diabetes, and cancer in particular. The occurrence of bleeding events was not significantly different across exposure groups. No significant differences across exposure groups existed in either the likelihood of incurring all-cause healthcare costs or in the magnitude of those costs. Warfarin-sensitive individuals were no more likely to utilize cardiovascular-related healthcare services; however, they had lower total and inpatient cardiovascular-related costs compared to warfarin-insensitive patients. No significant differences existed in any other categories of costs. We found limited evidence that warfarin-sensitive individuals have different healthcare spending than warfarin-insensitive individuals. Additional real-world studies are needed to support the traditional economic evaluations currently existing in the literature.

## Introduction

Oral anticoagulant (OAC) therapy has been the main treatment approach for stroke prevention for decades [1, 2]. OACs work by manipulating the blood coagulation process, thus thinning out an individual’s blood to prevent the formation of blood clots. Warfarin, a vitamin K antagonist, in particular, has been the most widely prescribed OAC in the United States and has been shown to be effective at reducing the risk of stroke in patients with non-valvular atrial fibrillation (NVAF) [3–5]. Despite the benefits of using warfarin to manage NVAF, it is not without its pitfalls. Warfarin treatment may be difficult to manage due to variability in dose requirements across individuals, as well as increased risk of bleeding, which results in a need for frequent monitoring of patients to ensure appropriate coagulation levels have been achieved and are being maintained [6–9].

Pharmacogenomics (PGx) studies how the presence of gene variants impacts an individual’s response to certain drugs [10]. This response may include the ability to metabolize the drug, the occurrence of drug-related adverse events, and/or effectiveness of the drug [11]. There are two genes associated with warfarin, *CYP2C9* and *VKORC1*. Individuals with common genetic variants in these genes (e.g., CYP2C9*2 and/or CYP2C9*3 for *CYP2C9* and *-1639G>A* for *VKORC1*) are at a greater risk for bleeding either due to a decreased ability to metabolize warfarin, as indicated by the *CYP2C9* gene, or due to a sensitivity to warfarin that requires reduced dosing, which is associated with the *VKORC1* variant [9]. Some individuals carry variants in both genes, further impacting their response to warfarin. Without properly adjusting dosing for patients with these gene variants, poor metabolizers and/or individuals with a sensitivity to warfarin may have higher rates of adverse drug events, more difficulty achieving their target anticoagulation levels, and as a result, have higher costs compared to those with a normal pharmacokinetic process [12–14]. PGx has the potential to mitigate risk concerns related to warfarin use by identifying these genetic variations associated with bleeding due to warfarin; thus fostering the opportunity to facilitate individualized medicine approaches to warfarin treatment, such as performing genome-guided dosing.

In order to evaluate the potential value of implementing PGx to ease the risk of treatment, it is important to first understand what differences exist between individuals with different genetic makeup related to the occurrence of clinical events (e.g., bleeding) and the utilization of healthcare services; and then to identify the financial implications of such differences. A systematic review conducted by Zhu et al (2019) summarized the empirical evidence on the cost effectiveness of implementing pharmacogenomics-guided treatment for cardiovascular related conditions [15]. Most of the studies included in this review examined the effects of pharmacogenomics testing, but virtually no studies looked at the impact of the actual genetic makeup of an individual on costs and outcomes.

This study aimed to assess whether differences in the prevalence of adverse bleeding outcomes exist between individuals with a normal ability to process warfarin (warfarin-insensitive) compared to a population of individuals with any type of genetic variant that would indicate a poor metabolic response or sensitivity to warfarin (warfarin-sensitive). We further aimed to assess whether any cost differences exist between these two groups.

## Methods

### Study population

The study population was derived from Mayo Clinic patients who are participants in The Right Drug, Right Dose, Right Time: Using Genomic Data to Individualize Treatment (RIGHT) 10K Study, which is an ongoing project aimed at studying the effects of “getting patients the right drug at the right dose at the right time based on their genetic information” [16, 17]. This study included RIGHT 10K patients receiving care at Mayo Clinic Rochester who received at least one prescription for warfarin (Coumadin/Jantoven) between 2004 and 2017. The date of the first available warfarin prescription for each patient during this time period was identified as that patient’s index date for this study. Any individuals who also received a prescription for warfarin (Coumadin/Jantoven) in the two years prior to their index date were excluded.

Data used in this study represented a combination of measures from Mayo Clinic’s billing and administrative data, as well as from the Mayo Clinic Rochester Cost Data Warehouse [18]. Persons in the RIGHT 10K study may receive some of their care from providers other than Mayo Clinic, and we recognize that we could miss some warfarin-related events. In addition, persons in the study who moved out of the area shortly after their index date would not have available outcome information in the Mayo Clinic medical records. Therefore, to account for these issues, patients who were empaneled to a Mayo Clinic provider for at least one year prior to their index date through one year post index and who also did not die in the year post were identified for subgroup analyses. Inclusion of empaneled patients to Mayo Clinic providers ensures that the subgroup analyses will have almost 100% of the healthcare utilizations at Mayo Clinic Rochester. This study was approved by Mayo Clinic’s Institutional Review Board.

### Exposure

Genome sequencing was completed for all subjects participating in the RIGHT 10K study. Genotyping data for genes *CYP2C9* and *VKORC1* were obtained from the RIGHT 10K study team and linked with our patient population. Phenotype interpretation for each individual gene, as well as a combined phenotype indicating the level of warfarin sensitivity of each patient was provided. The combined phenotype was broken into three levels of sensitivity: 1) those with a normal sensitivity to warfarin, 2) those with an intermediate sensitivity, and 3) those with a high sensitivity. These phenotypes are derived in alignment with the Clinical Pharmacogenetics Implementation Consortium guidelines for *CYP2C9* and *VKORC1* genotypes and warfarin dosing, as well as with the recommended daily warfarin doses outlined on the product insert for warfarin (Coumadin) approved by the US Food and Drug Administration [9, 19]. Our study combined those with an intermediate and high sensitivity into one exposure group. The reasons for this were two-fold: 1) due to the small sample size of the high sensitivity group and 2) to make inferences regarding costs and clinical outcomes relative to patients with any level of sensitivity versus those without.

### Outcomes

We examined two main outcomes of interest: 1) bleeding events and 2) healthcare costs. The year following each individual’s index date was examined to assess for bleeding events and total costs. Major bleeds were identified on the basis of ICD-9 and ICD-10 diagnosis codes present in any position on the claims billed during the follow-up year (S1 Appendix). Bleeding must have occurred in the following critical areas of the body, as outlined by the International Society of Thrombosis and Haemostatsis (ISTH) definition of major, overt bleeding: intracranial, intraspinal, intraocular, intra-articular, pericardial, retroperitoneal, or intramuscular with compartment syndrome [20]. In order for a bleeding event to be considered related to the receipt of warfarin, there must have also been an abnormal International Normalized Ratio (INR) value within the 2 weeks prior to or in the 2 weeks following the bleed.

Total healthcare costs were aggregated for the year following each individual’s index date. Standardized costs were obtained from the Mayo Clinic Rochester Cost Data Warehouse. Medicare reimbursement was assigned to all professional billed services, the appropriate Medicare Cost Report cost-to-charge ratios were multiplied by the charges for all hospital billed services, and all resulting costs were adjusted to 2017 dollars with the GDP Implicit Price Deflator [18]. In addition to aggregating total all-cause costs, we further identified a subset of cardiovascular-related costs (CV-related). We deemed costs to be CV-related if the primary diagnosis code on the claim could be linked to the Clinical Classification Software (CCS) diagnostic category for “Diseases of the Circulatory System” (CCS Multi-Level Category # 7) [21, 22]. Costs were further broken down by care delivery setting: Inpatient, Hospital Outpatient, Emergency Department (ED), and Clinic (e.g., office visit).

### Risk factor identification

We identified a number of baseline characteristics that had established clinical relevance to bleeding risk, and thus costs, or that would help control for patient complexity. We included such risk factors in our analysis to mitigate any confounding effects when examining bleeding and costs as outcomes. We determined which risk factors were relevant to our study through a systematic process. This included first developing conceptual models outlining the composition of risk factors at various levels of influence: the individual level, the interpersonal level, and the environmental level. Within each of these influence levels, specific risk factors were then identified using clinical reference sources [23–28]. Published literature was then searched to identify supporting empirical evidence for each risk factor. Finally, the magnitude of the effects found within the empirical evidence was recorded for consideration. Risk factors that were well cited with significant effects on warfarin side effects that were also readily available within our data sources were included for analysis.

The following risk factors were obtained for analysis: age, body mass index (BMI), number of prior bleeds, race, ethnicity, gender, education level, smoking status, and whether the individual received an alternative anticoagulant medication within the year prior to their index date. Demographic variables, such as birth date, race, ethnicity, gender, and education level, were extracted from Mayo Clinic’s self-reported registration data, using the most current record. Body mass index was extracted from the electronic medical record, taking the values recorded closest to the index date. Smoking status was obtained from previously recorded patient provided information using current visit information forms. Finally, prior bleeds and prior anticoagulation use were obtained using Mayo’s billing data and medication orders, respectively.

We also computed the Charlson comorbidity index with incorporated severity weighing to account for patient complexity [29–31]. Finally, we created additional disease indicator flags for our study population using diagnosis codes present on the claims in the year prior to each individual’s index date. We linked these baseline diagnosis codes to the CCS categories for easy identification of disease groups (S2 Appendix). Disease categories included were: hypertension, cardiovascular disease (CVD), myocardial infarction (MI), cerebrovascular disease, anemia, diabetes, malignancy, liver disease, diseases of the urinary system, thyroid disorders, mental illness, coagulation and hemorrhagic disorders.

### Analysis

We descriptively summarized cohort characteristics (e.g., patient demographics and risk factors) for our study population. We further compared these characteristics across exposure groups (e.g., warfarin-sensitive vs. warfarin-insensitive) and assessed for statistically significant differences using the Kruskal-Wallis test for continuous variables and the Chi-Square test for categorical variables. We used an alpha of 0.05 as the threshold for determining significance in all analyses. We also summarized the prevalence of specific gene variants present in our final cohort and additionally showed the distribution of the combined phenotypes within our sample.

We reported bleeding rates in the form of proportion with a bleed, as well as average number of bleeds, among those with and without sensitivity to warfarin. We further tested for differences in these measures across the two exposure groups using the Chi-Square test and Kruskal-Wallis test respectively. Average costs were reported and compared across exposure groups. Costs were assessed for differences in crude estimates using the Kruskal-Wallis test to account for the non-normal distribution of costs.

We used multivariate analysis to assess for significant differences in bleeding outcomes and costs across exposure groups while adjusting for important baseline characteristics and risk factors. Penalized maximum likelihood estimation methods described by Firth were used to model bleeding outcomes while taking into account the rarity of the events. [32, 33] Generalized linear models (GLMs) using a negative binomial distribution for overdispersed data were used to model the number of bleeding events.

Regular GLMs using a gamma distribution were used to model total all-cause costs and all-cause clinic-related costs due to a low prevalence of zero values. Conversely, models for all-cause inpatient, ED, and hospital outpatient costs, as well as all models for CV-related costs, were estimated using two-part regression models. A two-part regression model allows the researcher to account for large masses of observations at zero and for skewness in the remaining positive values by breaking down the modeling process into two components, with the first part modeling the probability of having a non-zero outcome and the second part modeling the outcome itself, conditional on that outcome being positive [34]. This approach is quite attractive for modeling healthcare costs, as costs are generally substantially skewed with a large number of observations with zero expenditures (e.g., individuals that don’t utilize healthcare services) [35]. Cost outcomes with a high prevalence of zero values were modeled using this two-part regression approach.

Model results for bleeding outcomes were reported as odds ratio and predicted probabilities (PP) for binary outcomes and as incident rate ratios and predicted average number of events for count outcomes. Predicted probabilities and predicted average costs (PAC) were reported for the likelihood of incurring costs (represented only in the two-part models) and for the magnitude of costs respectively. All data management and analysis were carried out using SAS 9.4 (SAS Institute Inc. Cary, NC).

## Results

### Cohort selection

**Fig 1:**
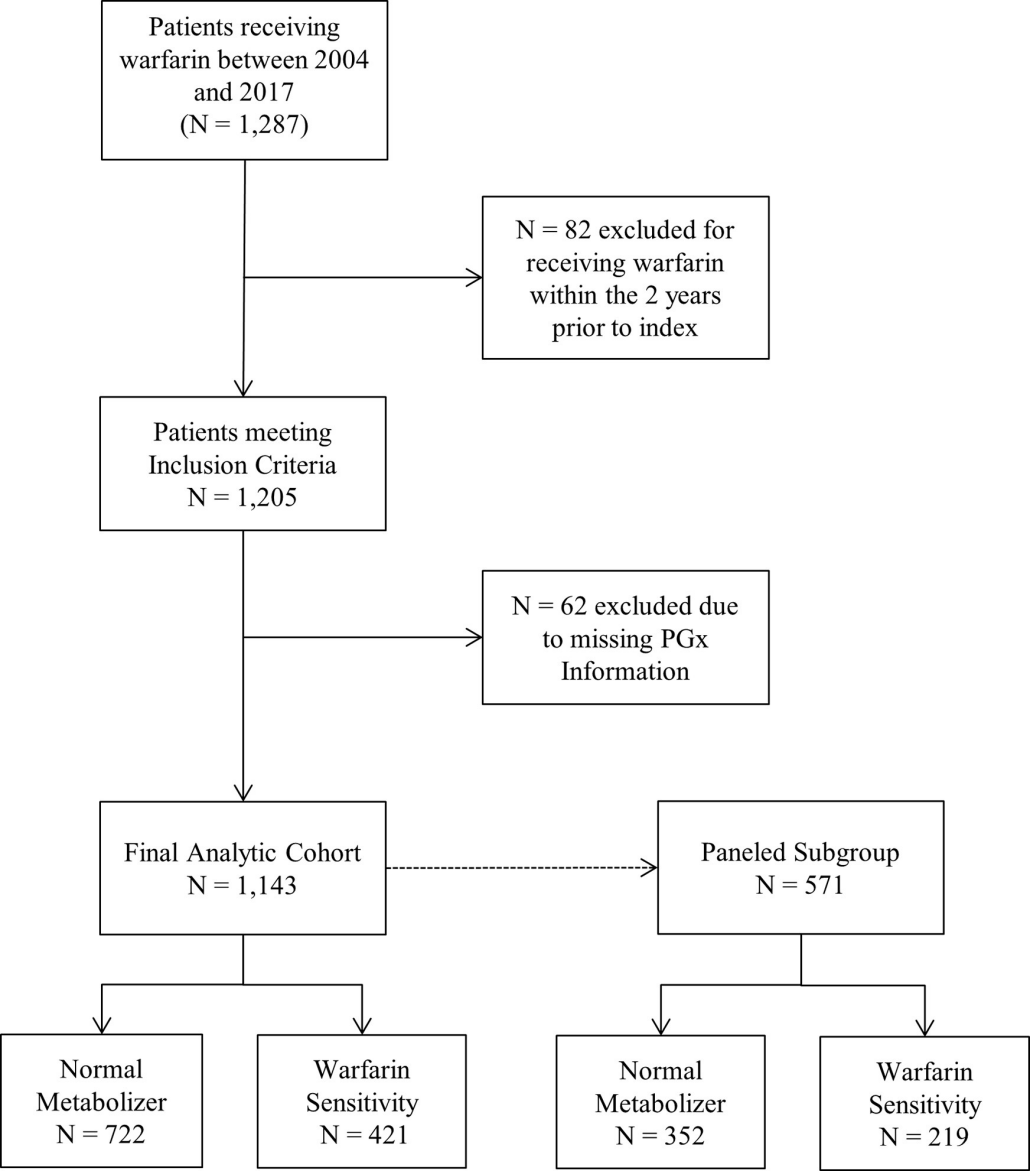
Flow diagram of cohort selection.

A total of N = 1,205 patients from the RIGHT 10K cohort met our study inclusion criteria (Fig 1). Of these patients, N = 62 (5.1%) did not have genotyping information available; thus their ability to metabolize warfarin was unknown. These patients were excluded from our analysis, leaving a final sample of N = 1,143. A total of N = 571 patients met the empanelment requirements to be analyzed as part of our subgroup analysis.

### Genotypes and phenotypes

In our final analytic sample of N = 1,143 patients, N = 720 (63.0%) had a “normal metabolizer” gene expression for *CYP2C9* (CYP2C9*1). The rest of our sample had some form of a genetic variant in the *CYP2C9* gene, with most having only one copy of the two most common variants, CYP2C9*1/*2 (20.3%) and CYP2C9*1/*3 (11.2%) (Table 1). Individuals with one of these gene variants, CYP2C9*2 and CYP2C9*3, and particularly those who have two copies of the variant (e.g., CYP2C9*2/*3) or also have a variant in the *VKORC1* gene, are those who do not metabolize warfarin as well as those with the wild-type genotype (i.e., CYP2C9*1/*1).

**Table 1:** Distribution of warfarin related genetic variants.

	Frequency
**CYP2C9 Genotype**	**N (%)**
1/1	720 (63.0%)
1/2	232 (20.3%)
1/3	128 (11.2%)
2/2	16 (1.4%)
2/3	22 (1.9%)
3/3	3 (0.3%)
Other Variant	22 (1.9%)
**Warfarin VKORC1 c.-1639 Genotype**	
A/A	149 (13.0%)
G/A	553 (48.4%)
G/G	437 (38.2%)
Other	4 (0.3%)
**Combined Phenotype**	
Normal Sensitivity to Warfarin	722 (63.2%)
Intermediate Sensitivity to Warfarin	385 (33.7%)
High Sensitivity to Warfarin	36 (3.1%)

Approximately 38% of individuals in our sample were homozygous carriers of the G allele in the *VKORC1* gen (i.e., *G/G*), indicating a normal genotype. Most of our sample (48.4%) carried the heterozygous *VKORC1* mutant genotype, *G/A*, while only 13.0% had the homozygous form of the mutation, *A/A* (Table 1). Consequently, the distribution of combined phenotypes in our study population was N = 722 (63.2%) with a normal sensitivity to warfarin, N = 385 (33.7%) with an intermediate sensitivity to warfarin, and N = 36 (3.1%) with a high sensitivity to warfarin. Thus, our exposure groups of interest included N = 421 warfarin-sensitive and N = 722 warfarin-insensitive patients.

The distribution of individual gene variants within the empaneled subgroup was virtually the same as that of the full population sample, although the subgroup had a slightly lower percentage of those with a normal sensitivity (61.6%), and a slightly higher percentage of those with an intermediate sensitivity (35.2%) (S1A Table). Within this group, there were N = 219 (38.4%) warfarin-sensitive and N = 352 (61.6%) warfarin-insensitive individuals (Fig 1).

### Baseline characteristics

Warfarin-sensitive patients in our sample were older, with a higher proportion of patients being 75 years or older (17.1% vs. 12.9%) and a lower proportion being less than 60 years old (19.0% vs 27.2%) compared to the warfarin-insensitive group (p = 0.01). Warfarin-sensitive individuals were also more likely to have a higher number of comorbidities compared to warfarin-insensitive individuals (Table 2, p = 0.008). Finally, warfarin-sensitive patients were more likely to have an MI in the year prior to their index (7.8% vs. 4.8%, p = 0.04), more likely to have a history of diabetes (33.5% vs. 28.0%, p = 0.05), and more likely to have cancer (39.9% vs. 32.8%, p = 0.02) when compared to warfarin-insensitive patients. There were no significant differences in the remaining baseline characteristics between exposure groups.

**Table 2:** Comparison of baseline characteristics across exposure groups.

Baseline Characteristic	Exposure Group		Total (N = 1,143)	P-Value
	Normal (N = 722)	Sensitive (N = 421)		
**Age**				0.01
<60	27.2%	19.0%	24.2%	
60–64	15.1%	16.9%	15.8%	
65–70	22.9%	26.4%	24.2%	
70–74	21.8%	20.5%	21.3%	
75+	12.9%	17.1%	14.5%	
**% Male**	55.1%	52.4%	54.1%	0.37
**% White**	97.9%	99.0%	98.3%	0.21
**% Hispanic**	1.9%	1.2%	1.7%	0.34
**BMI**				0.91
Underweight	0.6%	0.5%	0.5%	
Normal	12.0%	10.5%	11.5%	
Overweight	25.6%	27.1%	26.2%	
Obese	42.4%	41.6%	42.1%	
Unknown	19.4%	20.4%	19.8%	
**Education Level**				0.55
Some high school	0.8%	0.7%	0.8%	
High school/GED	16.9%	21.0%	18.4%	
Some college or 2 yr degree	30.7%	30.0%	30.4%	
4 yr college degree	16.4%	16.0%	16.2%	
Post graduate studies	35.1%	32.4%	34.1%	
**Smoking Status**				0.25
Non-User	55.8%	52.5%	54.6%	
Current User	15.1%	19.7%	16.8%	
Former User	22.0%	20.9%	21.6%	
Unknown	7.1%	6.9%	7.0%	
**Charlson Index**				0.008
0	40.9%	35.2%	38.8%	
1	22.2%	18.5%	20.8%	
2	14.5%	14.0%	14.3%	
3	9.4%	13.8%	11.0%	
4 or more	13.0%	18.5%	15.0%	
**% with any prior bleed**	25.2%	25.9%	25.5%	0.80
**% received anticoagulant in year prior**	8.4%	7.4%	8.0%	0.51
**Hypertension**	54.0%	56.8%	55.0%	0.37
**Cardiovascular Disease**	63.4%	61.8%	62.8%	0.57
**Myocardial Infarction**	4.8%	7.8%	5.9%	0.04
**Cerebrovascular Disease**	8.3%	9.3%	8.7%	0.58
**Anemia**	19.1%	21.1%	19.9%	0.41
**Diabetes**	28.0%	33.5%	30.0%	0.05
**Malignancy**	32.8%	39.9%	35.4%	0.02
**Liver disease**	7.3%	9.3%	8.0%	0.25
**Diseases of the Urinary System**	31.2%	30.6%	31.0%	0.85
**Thyroid Disorders**	13.4%	16.4%	14.5%	0.17
**Mental Illness**	27.7%	30.4%	28.7%	0.33
**Coagulation and Hemorrhagic Disorders**	6.6%	6.9%	6.7%	0.88

When examining the empaneled subgroup population, the only significant difference in risk factors between exposure groups that existed was in the Charlson index. Similar to the full population, warfarin-sensitive individuals within the empaneled subgroup were more likely to have a higher number of comorbid conditions, with 13.2% having 3 conditions and 18.7% having four or more conditions compared to 8.0% and 13.6% respectively among warfarin-insensitive (p = 0.03) (S1B Table).

### Crude outcomes

There were N = 97 (8.5%) patients who experienced a major bleed in the year after their index warfarin date. There was no significant difference (p = 0.87) in the proportion of subjects with bleeding events across exposure groups, with 35 (8.3%) subjects with bleeds in the warfarin-sensitive group and 62 (8.6%) subjects with bleeds in the warfarin-insensitive group. Among those with bleeds, warfarin-sensitive patients had an average of 4.26 (SD = 4.01) bleeding events per person in the year following their index date compared to an average of 3.47 (SD = 2.58) bleeding events per person in the warfarin-insensitive group, although this difference was not statistically significant (p = 0.70; Table 3). Among the empaneled subgroup population, 52 (9.1%) patients had a major bleed in the year following their index. Similar to the full population, there were no significant differences in the proportion of individuals with bleeds (9.13% vs. 9.09%; p = 0.99) or in the average number of bleeds among those with bleeds (4.5 vs. 3.44; p = 0.41) when comparing warfarin-sensitive to warfarin-insensitive patients (S1C Table).

**Table 3:** Summary of crude outcomes across exposure groups.

	Normal (N = 722)		Sensitive (N = 421)		P-Value
	Mean (SD)	Median (Q1,Q3)	Mean (SD)	Median (Q1,Q3)	
**Bleeding Events (#/patient)**	3.47 (2.58)	2 (2,5)	4.26 (4.01)	3 (1,5)	0.70 (0.78)
**All-Cause Costs ($)**					
Total Costs	$17,150.11 ($27,963.82)	$9,167.76 ($3,554.91, $21,055.51)	$17,570.73 ($36,407.37)	$8,574.76 ($3,559.27, $19,070.51)	0.64(0.49)
Inpatient Costs	$10,761.70 ($23,959.72)	$677.65 ($0.00, $15,472.50)	$10,774.82 ($31,590.13)	$677.43 ($0.00, $14,703.38)	0.80 (0.79)
Emergency Department Costs	$255.01 ($797.12)	$0.00 ($0.00, $0.00)	$217.70 ($729.66)	$0.00 ($0.00, $0.00)	0.47 (0.99)
Hospital Outpatient Costs	$3,163.65 ($6,620.98)	$765.17 ($0.00, $3,694.38)	$3,523.49 ($8,970.38)	$803.17 ($0.00, $3,662.45)	0.64 (0.91)
Clinic Costs	$2,969.75 ($3,212.83)	$2,277.85 ($1,144.23, $3,927.65)	$3,054.72 ($3,411.67)	$2,331.69 ($1,199.85, $3,906.69)	0.68 (0.68)
**CV-Related Costs ($)**					
Total Costs	$5,689.24 ($18,660.11)	$677.30 ($22.51, $3,234.14)	$4,551.59 ($21,545.39)	$684.37 ($56.33, $3,123.32)	0.89 (0.78)
Inpatient Costs	$3,637.72 ($16,863.40)	$0.00 ($0.00, $143.84)	$2,728.13 ($20,556.82)	$0.00 ($0.00, $190.57)	0.71 (0.99)
Emergency Department Costs	$76.65 ($416.68)	$0.00 ($0.00, $0.00)	$62.50 ($329.89)	$0.00 ($0.00, $0.00)	0.77 (1.00)
Hospital Outpatient Costs	$1,235.53 ($3,702.90)	$0.00 $(0.00, $548.03)	$1,046.31 ($2,935.38)	$0.00 ($0.00, $475.38)	0.61 (1.00)
Clinic Costs	$739.34 ($1,072.66)	$384.99 ($11.18, $1,044.69)	$714.65 ($993.06)	$355.37 ($43.82, $991.42)	0.71 (0.37)

Among the warfarin-sensitive group, the majority of costs incurred were from inpatient services (61%), followed by hospital outpatient (20%) and clinic costs (17%), with a small proportion of total costs being from emergency department services (2%). Comparable findings were present among the warfarin-insensitive group with proportions of 63%, 18%, 17%, and 2% respectively. CV-related costs followed the same distributional pattern, with the exception of hospital outpatient services having a higher share of the distribution and clinic costs having a lower share of the distribution. There were no significant differences in unadjusted average costs between the two exposure groups (Table 3).

### Multivariate analysis

We identified a number of baseline characteristics and risk factors to potentially adjust for in our multivariate analysis, including age, BMI, ethnicity, gender, education level, smoking status, history of prior bleeding, prior use of an alternative anticoagulant, and a number of comorbid conditions either included as part of the Charlson index or as one of our risk factors of interest. In order to conserve power given our small sample size, we identified a more parsimonious model to report on which included the following risk factors: age, Charlson index, history of myocardial infarction, history of diabetes, and history of cancer. These factors were chosen based on whether they were significantly associated with the exposure of interest (warfarin sensitivity) on a univariate basis.

We detected no significant effect of warfarin sensitivity on the probability of experiencing a major bleeding event (Sensitive: PP = 0.08 vs. Insensitive: PP = 0.09, OR = 0.84, p = 0.44). Furthermore, there was no significant effect on the average number of bleeding events among the full study population when comparing sensitive to not sensitive individuals (PP: 0.24 vs. 0.27, IRR = 0.90, p = 0.74). We further subset the sample to only those individuals who experienced a bleed, yet were still unable to detect a significant effect on the average number of bleeding events (PP: 3.92 vs. 3.45, IRR = 1.14, p = 0.43) (Table 4). No differing results were found among the empaneled subgroup population (S1D Table).

**Table 4:** Predicted probabilities of experiencing a major bleeding event and predicted average number of bleeding events across exposure groups for warfarin sensitivity^a^.

	Regression Estimates (odds ratio and incident rate ratios)	Predictive Margins (predicted probabilities of predicted average number of events)		
Outcome	Sensitive vs. Normal	Normal	Sensitive	P-Value
**Experiencing a Major Bleeding Event**				0.44
Estimate	0.84	0.09	0.08	
95% CI	(0.54, 1.31)	(0.07, 0.12)	(0.05, 0.11)	
**Number of Major Bleeding Events (entire study sample)**				0.74
Estimate	0.90	0.27	0.24	
95% CI	(0.48, 1.70)	(0.17, 0.36)	(0.12, 0.36)	
**Number of Major Bleeding Events (for those who experienced a bleed)**				0.43
Estimate	1.14	3.45	3.92	
95% CI	(0.83, 1.55)	(2.80, 4.10)	(2.96, 4.88)	

^a^ Each estimated was generated from a multivariate regression model regressing warfarin sensitivity on each of the bleeding outcomes, controlling for the following adjusting variables: age, Charlson index, history of myocardial infarction, history of diabetes, and history of cancer.

Examination of adjusted standardized all-cause costs revealed no significant differences across exposure groups in either the likelihood of incurring healthcare costs or in the magnitude of costs (Table 5). This finding was true both for total all-cause costs (p = 0.32), as well as costs subset into each of the different care delivery settings (inpatient: p = 0.91 and p = 0.36; ED: p = 0.49 and p = 0.33; hospital outpatient: p = 0.76 and p = 0.91; clinic: p = 0.84).

**Table 5:** Predicted probabilities of incurring costs and predicted average costs across exposure groups for warfarin sensitivity^a^.

	Predicted Probabilities of Incurring Costs (Logit Model^b^)		Predicted Mean Costs (Gamma Model^c^)		Combined–Predicted Mean Costs (Two-Part Model)	
	Mean (95% CI)		Mean (95% CI)		Mean (95% CI)	
Outcome	Normal	Sensitive to Warfarin	Normal	Sensitive to Warfarin	Normal	Sensitive to Warfarin
**All Cause**						
Total Costs			$17,791.67 ($15,813.60, $19,769.73)	$16,547.75 ($14,275.49, $18,820.01)		
Inpatient Costs	0.66 (0.63, 0.70)	0.67 (0.62, 0.71)	$16,983.20 ($14,398.93, $12,458.68)	$15,386.72 ($12,458.68, $18,314.76)	$11,157.28 ($9,364.18, $12,950.38)	$10,155.09 ($8,110.36, $12,199.82)
Emergency Department Costs	0.17 (0.14, 0.19)	0.15 (0.12, 0.018)	$1,508.83 ($1,274.39, $1,743.27)	$1,342.79 ($1,058.67, $1,626.92)	$260.71 ($202.16, $319.26)	$210.79 ($146.11, $275.47)
Hospital Outpatient Costs	0.62 (0.59, 0.66)	0.63 (0.58, 0.68)	$5,289.15 ($4,534.25, $6,044.03)	$5,343.43 ($4,392.09, $6,294.76)	$3,265.22 ($2,764.69, $2,765.75)	$3,343.39 ($2,704.51, $3,982.28)
Clinic Costs			$3,024.13 ($2,820.48, $3,227.77)	$2,989.05 ($2,735.08, $3,243.03)		
**CV-Related**						
Total Costs	0.82 (0.79, 0.85)	0.82 (0.78, 0.86)	$7,761.07*** ($5,820.59, $9,701.54)	$5,196.72*** ($3,670.54, $6,722.91)	$6,151.05*** ($4,596.02, $7,706.07)	$4,142.41*** ($2,914.374, $5,370.44)
Inpatient Costs	0.27 (0.24, 0.30)	0.27 (0.23, 0.31)	$14,126.55* ($8,800.09, $19,453.01)	$8,723.65* ($4,746.79, $12,700.52)	$4,031.26* ($2,374.72, $5,687.80)	$2,485.36* ($1,293.76, $3,676.96)
Emergency Department Costs	0.04 (0.03, 0.06)	0.04 (0.02, 0.06)	$1,519.81 ($1,201.46, $1,838.15)	$1,281.88 ($917.53, $1,646.24)	$75.48 ($46.13, $104.83)	$64.03 ($30.63, $97.43)
Hospital Outpatient Costs	0.36 (0.32, 0.39)	0.33 (0.28, 0.37)	$3,296.67 ($2,656.53, $3,936.81)	$3,265.46 ($2,364.36, $4,166.56)	$1,192.58 ($933.03, $1,452.13)	$1,084.66 ($750.39, $1,418.93)
Clinic Costs	0.79 (0.76, 0.82)	0.81 (0.78, 0.85)	$965.86 ($876.77, $1,054.95)	$892.58 ($787.85, $997.30)	$744.31 ($669.87, $818.74)	$710.30 ($620.11, $800.49)

^a^ Each estimated was generated from a multivariate regression model regressing warfarin sensitivity on each of the cost categories, controlling for the following adjusting variables: age, Charlson index, history of myocardial infarction, history of diabetes, and history of cancer.

^b^ Rows that have estimates populated for the Logit model were run using a two-part model.

^c^ Rows that only have estimates for the Gamma model were run with a regular generalized linear model using a gamma distribution to model costs.

* represents significance at the <0.05 level.

** represents significance at the <0.01 level.

** represents significance at the <0.001 level.

Results from the empaneled subgroup population were similar to those of the full sample. Models for the subgroup were estimated using the same approaches as those used on the full population sample. There were no significant differences across exposure groups in total costs (p = 0.90) or in any individual component of cost (inpatient: p = 0.34 and p = 0.70; ED: p = 0.96 and p = 0.65; hospital outpatient: p = 0.99 and p = 0.68; clinic: p = 0.73) after adjusting for baseline covariates (S1E Table).

We also performed multivariate adjusted regression analysis on CV-related costs (Table 5). There was no significant difference in the likelihood of generating total CV-related costs (PP: 0.82 vs. 0.82; OR = 1.04; p = 0.82); however, among those who spend something, the GLM model shows that warfarin-sensitive patients had less total CV-related costs (PAC: $5,196.72) when compared to warfarin-insensitive patients (PAC: $7,761.07, p = 0.0005). For the overall exposure effect combining both parts of the two-part model, we find that warfarin-sensitive individuals spend less on total CV-related services than warfarin-insensitive individuals ($4,142.41 vs. $6,151.05). Similar findings were present when examining CV-related inpatient costs for both the logit model for incurring costs (PP: 0.27 vs. 0.27; OR = 0.99, p = 0.99), as well as the GLM model (PAC: $8,723.65 vs. $14,126.55, p = 0.0213) and the combined effects (PAC: $2,485.36 vs. $4,031.26). There were no significant differences in the likelihood of generating CV-related ED, hospital outpatient, or clinic costs. Likewise, there were no significant differences in the magnitude of CV-related costs across all care settings for either the GLM model or the combined effect of the two-part model.

Among the empaneled subgroup of patients, there was no significant difference in the likelihood of incurring total CV-related costs (p = 0.512); however, similar to the full population sample, among those who spent something, warfarin-sensitive individuals were found to have significantly lower total CV-related costs than warfarin-insensitive (PAC: $4,676.83 vs. $7,255.25; p = 0.045), which was also present in the combined effect (PAC: $3,254.69 vs. $5,187.78). We also found that both groups were equally likely to generate costs within each individual care setting and that there were no significant differences between the two exposure groups in the magnitude of costs incurred for either the GLM model or the combined effect of the two-part model (S1E Table). Complete regression results are available for both the full study population and the empaneled subgroup in S2 Table and S3 Table, respectively.

## Discussion

Our study examined differences in baseline characteristics, bleeding outcomes, and costs between those with a normal drug response to warfarin and a group of individuals with genetic variants that are likely to create warfarin sensitivity or facilitate poor metabolization of warfarin, thus increasing the risk of adverse events among this population. We found that just over a third of our study population (34.9%) carried a warfarin-sensitive phenotype. Existing literature shows that approximately two-thirds of individuals in the Caucasian population have the wild type genotype (i.e., CYP2C9*1/*1) [36–39]. Given that our study population is predominately white, with a low rate of Hispanic individuals, our findings are consistent with the current literature. A handful of studies also displayed slightly higher or slightly lower rates of gene mutations in a Caucasian population, demonstrating that the generalizability of our findings may vary across population samples [40, 41].

Warfarin-sensitive individuals differed in their baseline characteristics by being of older age and having a higher number of comorbid conditions; MI, diabetes, and cancer in particular. The prevalence of bleeding events, both in terms of the number of individuals experiencing a bleed, as well as the average number of bleeds per person, was not significantly different across exposure groups. These results are consistent with some published studies examining the influence of *CYP2C9* and *VKORC1* polymorphisms on clinical outcomes, but empirical evidence is mixed. Studies by Higashi et al. and Margaglione et al. demonstrated a clear increased risk of bleeding among individuals with a variant genotype [42, 43]. Meckley et al. also demonstrated an increased risk of bleeding for those with a variant in the *CYP2C9* gene, but not for *VKORC1* [44]. Research by Limdi et al., Jorgensen et al., and Samardzija et al. showed only marginal differences in bleeding risk or found crude differences that did not retain significance once analyses were appropriately adjusted [45–47]. A number of other papers, similar to our study, found no significant effects of the *CYP2C9* or *VKORC1* genes on risk of bleeding [48–51].

Warfarin-sensitive individuals were no more likely to utilize healthcare services, and thus did not incur higher costs compared to those warfarin-insensitive individuals. In terms of the magnitude of costs, generalized linear and two-part regression models revealed that warfarin-sensitive individuals were likely to have lower total CV-related costs when compared to those with a normal drug response to warfarin. This finding is likely driven by the fact that warfarin-sensitive individuals were also likely to have lower inpatient CV-related costs than warfarin-insensitive individuals. There were no significant differences in any other categories of cost.

Lower costs among the warfarin-sensitivite population may be reflective of better care management due to the presence of more comorbid conditions. These individuals may concurrently be receiving healthcare services to manage one or more of their other diseases, which may impact their OAC treatment approach. Furthermore, our study results may be reflective of the nature of our sample population, as patients identified for this study were individuals enrolled in the Mayo Clinic Biobank, in addition to being RIGHT 10K study participants. Thus, they may represent a slightly different population of individuals who are engaged in health care and are interested in examining how their own genetics and can impact health and well-being. Voluntary enrollment in such initiatives may influence health outcomes as well. Furthermore, because the Mayo Clinic Biobank is not population based, the pool of participants may not be completely representative of the general population. According to Olson et al, the Biobank population reflects a more highly educated group of individuals as compared to national rates or even Olmsted County residents in general [52].

Ours is not the first study that was unable to discern a clear value of pharmacogenomics in real world practice. A study by Billings et al. used a real world cohort to evaluate the cost effectiveness of genotype guided warfarin therapy versus use of an alternative treatment option [53]. The study found that individuals who received PGx testing had higher spending for both all-cause and cardiovascular disease related costs versus those who did not have testing completed, leading the authors to conclude that the value of PGx for warfarin is questionable. Furthermore, a systematic review by Zhu et al demonstrated that almost half of studies examining the cost effectiveness of PGx testing for warfarin conclude no cost effectiveness or mixed results [15]. Most existing studies examining this question rely on simulation modeling with hypothetical cohorts. Few studies use real world cohorts and typically examine PGx tested versus non-tested individuals rather than sensitive versus insensitive exposure groups based on genotype. These facts make our study a novel addition to the literature.

Mayo Clinic is a large integrated delivery network, making it possible for patients to receive the full spectrum of healthcare services through a single organization. We consider this to be a strength, despite being a single center study, as we anticipated limited loss to follow up for this particular population of interest being that they were RIGHT 10K study participants. Use of an empaneled subgroup to further ensure full capture of healthcare services helped corroborate our findings of the full population sample. Additional strengths of our study include thoughtful application of stratification methods and varied definitions of bleeding outcomes, which may allow for more widely applicable findings. Finally, because the intent of this research is to inform clinicians about the potential value of pharmacogenomics, we were rigorous in our outcomes definitions and chose to pursue both a clinical based outcome (bleeding) and an economic outcome (cost) to provide a well-rounded analysis for decision making.

Our study was subject to some limitations. First, we were constrained in our ability to detect meaningful differences due to our small sample size. We also identified warfarin initiation based on medication orders. Thus, we were not able to ascertain whether an individual actually took and adhered to the treatment of interest, which could impact cost and bleeding outcomes. Although we were fairly inclusive in our population definition and considered a wide variety of confounding factors to reduce bias, there may still be residual bias present in our study. For example, we were not able to identify individuals with an allergy or previous reaction to warfarin. These individuals would be unlikely to receive warfarin going forward, and thus would not be included in our study. Additionally, direct oral anticoagulants were introduced at the mid-point of our study period, at which point warfarin began to be progressively substituted with these new therapies over time [54]. Thus, our study population, particularly individuals initiating warfarin in or after 2010, represents a subset of all individuals receiving anticoagulant therapy during this time. This fact contributed to the risk of bias in our study. Finally, our study is constrained due to limitations inherent in using claims or billing data for analysis (e.g., miscoding, coding for payment rather than research purposes, only capturing things that generate a bill, etc.). These limitations may impact the generalizability of our study.

## Conclusions

While a number of economic evaluations exist examining the cost-effectiveness of pharmacogenomics testing for warfarin, few real-world observational studies exist to support these studies, and even fewer use genotype as the main exposure of interest. Our study examined a cohort of individuals initiating warfarin therapy and examined bleeding and cost outcomes for the year following initiation. Our study found limited evidence that individuals who are poor metabolizers or have a sensitivity to warfarin have different patterns of healthcare spending than those who have a normal drug response to warfarin; however, we were limited in our findings due to a small sample size. Considering the already mixed evidence on pharmacogenomics testing for warfarin, additional real-world studies are needed to support the evidence generated by the traditional economic evaluations that currently exist in the literature.

## Supporting information

S1 Appendix ICD-9 and ICD-10 codes to identify bleeding events. This table contains to complete list if ICD-9 and ICD-10 diagnosis codes that were used to identify bleeding events of interest from Mayo Clinic billing data.
(DOCX)

S2 Appendix CCS categories used to identify risk factors. This table contains the standard Clinical Classification Software categories used from the Healthcare Cost and Utilization Project to aid in the identification of important risk factors of interest.
(DOCX)

S1 Table Supplemental results tables for empaneled subgroup. These tables include the primary results (Tables 1 through 5) for the subgroup analysis performed on the empaneled patient population.
(DOCX)

S2 Table Complete regression results for the full study population. These tables contain the full regression results (parameter estimates, standard errors, 95% confidence intervals, and p-values for all covariates included in the multivariate models), with models being performed on the full study population. A separate table for each regression outcome of interest is included.
(DOCX)

S3 Table Complete regression results for the empaneled subgroup. These tables contain the full regression results (parameter estimates, standard errors, 95% confidence intervals, and p-values for all covariates included in the multivariate models), with models being performed on the empaneled subgroup. A separate table for each regression outcome of interest is included.
(DOCX)
