# Supplementary material for: Comparing outcomes and costs among warfarin-sensitive patients versus warfarin-insensitive patients using The Right Drug, Right Dose, Right Time: Using genomic data to individualize treatment (RIGHT) 10K warfarin cohort
Source: PLoS One. 2020 May 19;15(5):e0233316. doi: 10.1371/journal.pone.0233316 (PMC7237006; doi:10.1371/journal.pone.0233316)
Supplement: S1 Appendix — This table contains to complete list if ICD-9 and ICD-10 diagnosis codes that were used to identify bleeding events of interest from Mayo Clinic billing data. (DOCX) [file pone.0233316.s001.docx]

**S1 Appendix. ICD-9 and ICD-10 codes to identify bleeding events**

| **ICD Code** | **ICD Code Type** | **ICD Code Description** |
| --- | --- | --- |
| 360.43 | ICD-9 | Hemophthalmos, except current injury |
| 362.43 | ICD-9 | hemorrhagic detachment of retinal pigment epithelium |
| 362.81 | ICD-9 | retinal hemorrhage |
| 363.6 | ICD-9 | choroidal hemorrhage and rupture |
| 363.61 | ICD-9 | choroidal hemorrhage |
| 363.62 | ICD-9 | expulsive choroidal hemorrhage |
| 363.72 | ICD-9 | hemorrhagic choroidal detachment |
| 364.41 | ICD-9 | hyphema of iris and ciliary body |
| 372.72 | ICD-9 | conjuctival hemorrhage |
| 374.81 | ICD-9 | hemorrhage of eyelid |
| 376.32 | ICD-9 | orbital hemorrhage |
| 377.42 | ICD-9 | hemorrhage in optic nerve sheaths |
| 379.23 | ICD-9 | vitreous hemorrhage |
| 423.0 | ICD-9 | Hemopericardium |
| 430 | ICD-9 | subarachnoid hemorrhage |
| 431 | ICD-9 | intracerebral hemorrhage |
| 432 | ICD-9 | intracranial hem nec/nos |
| 432.0 | ICD-9 | intracranial hemorrhage |
| 432.1 | ICD-9 | subdural hemorrhage |
| 432.9 | ICD-9 | intracranial hemorr NOS |
| 455.2 | ICD-9 | int hemrrhoid w comp nec |
| 455.5 | ICD-9 | ext hemrrhoid w comp nec |
| 455.8 | ICD-9 | hemrrhoid nos w comp nec |
| 456.0 | ICD-9 | esophag varices w bleed |
| 456.20 | ICD-9 | esoph varices in oth diseases with bleeding |
| 459.0 | ICD-9 | hemorrhage nos |
| 530.21 | ICD-9 | Ulcer of esophagus with bleeding |
| 530.7 | ICD-9 | mallory-weiss syndrome |
| 530.82 | ICD-9 | esophageal hemorrhage |
| 531 | ICD-9 | upper gastrointestinal bleed |
| 531.0 | ICD-9 | ac stomach ulcer w hem |
| 531.00 | ICD-9 | ac stomach ulcer w hem |
| 531.01 | ICD-9 | ac stomach ulcer w hem-obst |
| 531.2 | ICD-9 | ac stomach ulc w hem/perf |
| 531.20 | ICD-9 | ac stomach ulc w hem/perf |
| 531.21 | ICD-9 | ac stomach ulc w hem/perf-obst |
| 531.4 | ICD-9 | chr stomach ulc w hem |
| 531.40 | ICD-9 | chr stomach ulc w hem |
| 531.41 | ICD-9 | chr stomach ulc w hem-obst |
| 531.6 | ICD-9 | chr stomach ulc hem/perf |
| 531.60 | ICD-9 | chr stomach ulc hem/perf |
| 531.61 | ICD-9 | chr stomach ulc hem/perf-obst |
| 532 | ICD-9 | upper gastrointestinal bleed |
| 532.0 | ICD-9 | ac duodenal ulcer w hem |
| 532.00 | ICD-9 | ac duodenal ulcer w hem |
| 532.01 | ICD-9 | ac duodenal ulcer w hem-obst |
| 532.2 | ICD-9 | ac duoden ulc w hem/perf |
| 532.20 | ICD-9 | ac duodenal ulc w hem/perf |
| 532.21 | ICD-9 | ac duodenal ulc w hem/perf-obst |
| 532.4 | ICD-9 | chr duoden ulcer w hem |
| 532.40 | ICD-9 | chr duoden ulcer w hem |
| 532.41 | ICD-9 | chr duoden ulcer w hem-obst |
| 532.6 | ICD-9 | chr duoden ulc hem/perf |
| 532.60 | ICD-9 | chr duoden ulc w hem/perf |
| 532.61 | ICD-9 | chr duoden ulc w hem/perf-obst |
| 533 | ICD-9 | peptic ulcer |
| 533.0 | ICD-9 | ac peptic ulcer w hemorr |
| 533.00 | ICD-9 | ac peptic ulc w hemorr |
| 533.01 | ICD-9 | ac peptic ulc w hemorr-obst |
| 533.2 | ICD-9 | ac peptic ulc w hem/perf |
| 533.20 | ICD-9 | ac peptic ulc w hem/perf |
| 533.21 | ICD-9 | ac peptic ulc w hem/perf-obst |
| 533.4 | ICD-9 | chr peptic ulcer w hem |
| 533.40 | ICD-9 | chr peptic ulcer w hem |
| 533.41 | ICD-9 | chr peptic ulcer w hem-obst |
| 533.6 | ICD-9 | chr pept ulc w hem/perf |
| 533.60 | ICD-9 | chr peptic lc w hem/perf |
| 533.61 | ICD-9 | chr peptic lc w hem/perf-obst |
| 534 | ICD-9 | upper gastrointestinal bleed |
| 534.0 | ICD-9 | ac marginal ulcer w hem |
| 534.00 | ICD-9 | ac marginal ulcer w hem |
| 534.01 | ICD-9 | ac marginal ulcer w hem-obst |
| 534.2 | ICD-9 | ac margin ulc w hem/perf |
| 534.20 | ICD-9 | ac margin ulc w hem/perf |
| 534.21 | ICD-9 | ac margin ulc w hem/perf-obst |
| 534.4 | ICD-9 | chr marginal ulcer w hem |
| 534.40 | ICD-9 | chr marginal ulcer w hem |
| 534.41 | ICD-9 | chr marginal ulcer w hem-obst |
| 534.6 | ICD-9 | chr margin ulc hem/perf |
| 534.60 | ICD-9 | chr marg ulc w hem/perf |
| 534.61 | ICD-9 | chr marg ulc w hem/perf-obst |
| 535.01 | ICD-9 | acute gastritis with hemorrhage |
| 535.11 | ICD-9 | atrophic gastritis with hemorrhage |
| 535.21 | ICD-9 | gastr mucosoal hypertroph with hemorrhage |
| 535.31 | ICD-9 | alcoholic gastritis with hemorrhage |
| 535.41 | ICD-9 | gastritis nec with hemorrhage |
| 535.51 | ICD-9 | gastritis/duodenitis nos with hemorrhage |
| 535.61 | ICD-9 | duodenitis with hemorrhage |
| 537.83 | ICD-9 | angiodysplasia of stomach and duodenum with hemorrhage |
| 537.84 | ICD-9 | dieulafoy lesion (hemorrhagic) of stomach and duodenum |
| 562.02 | ICD-9 | diverticula sm intestine w hemorrhage |
| 562.03 | ICD-9 | diverticulitis sm intestine w hemorrhage |
| 562.12 | ICD-9 | diverticula of colon w hemorrhage |
| 562.13 | ICD-9 | diverticulitis of colon w hemorrhage |
| 568.81 | ICD-9 | hemoperitoneum |
| 569.3 | ICD-9 | rectal & anal hemorrhage |
| 569.85 | ICD-9 | angiodysplasia with hem nec |
| 569.86 | ICD-9 | gastrointestinal bleeding |
| 578 | ICD-9 | gastrointestinal hemorr |
| 578.0 | ICD-9 | hematemesis |
| 578.1 | ICD-9 | blood in stool |
| 578.9 | ICD-9 | hemorrhage of gastrointestinal tract, unspecified |
| 596.7 | ICD-9 | hemorrhage into bladder wall |
| 599.7 | ICD-9 | hematuria |
| 599.70 | ICD-9 | hematuria |
| 599.71 | ICD-9 | gross hematuria |
| 719.10 | ICD-9 | hemarthrosis |
| 719.1 | ICD-9 | other hemorrhage |
| 719.11 | ICD-9 | hemarthrosis |
| 719.12 | ICD-9 | hemarthrosis |
| 719.13 | ICD-9 | hemarthrosis |
| 719.14 | ICD-9 | hemarthrosis |
| 719.15 | ICD-9 | hemarthrosis |
| 719.16 | ICD-9 | hemarthrosis |
| 719.17 | ICD-9 | hemarthrosis |
| 719.18 | ICD-9 | hemarthrosis |
| 719.19 | ICD-9 | hemarthrosis |
| 784.7 | ICD-9 | epistaxis |
| 784.8 | ICD-9 | hemorrhage from throat |
| 786.3 | ICD-9 | hemoptysis |
| 786.30 | ICD-9 | hemoptysis, unspecified |
| 998.11 | ICD-9 | hemorrhage complicating a procedure |
| D78.01 | ICD-10 | intraoperative hemorrhage and hematoma of the spleen complicating a procedure on the spleen |
| D78.02 | ICD-10 | intraoperative hemorrhage and hematoma of the spleen complicating other procedure |
| D78.21 | ICD-10 | postprocedural hemorrhage and hematoma of the spleen following a procedure on the spleen |
| D78.22 | ICD-10 | postprocedural hemorrhage and hematoma of the spleen following other procedure |
| E36.01 | ICD-10 | intraoperative hemorrhage and hematoma of an endocrine system organ or structure complicating an endocrine system procedure |
| E36.02 | ICD-10 | intraoperative hemorrhage and hematoma of an endocrine system organ or structure complicating other procedure |
| E89.81 | ICD-10 | Postprocedural hemorrhage of an endocrine system organ or structure following a procedure |
| E89.810 | ICD-10 | Postprocedural hemorrhage of an endocrine system organ or structure following an endocrine system procedure |
| E89.811 | ICD-10 | Postprocedural hemorrhage of an endocrine system organ or structure following other procedure |
| G97.31 | ICD-10 | intraoperative hemorrhage and hematoma of a nervous system organ or structure complicating a nervous system procedure |
| G97.32 | ICD-10 | intraoperative hemorrhage and hematoma of a nervous system organ or structure complicating other procedure |
| G97.51 | ICD-10 | postprocedural hemorrhage and hematoma of a nervous system organ or structure following a nervous system procedure |
| G97.52 | ICD-10 | postprocedural hemorrhage and hematoma of a nervous system organ or structure following other procedure |
| H02.89 | ICD-10 | other specified disorders of eyelid |
| H05.23 | ICD-10 | Hemorrhage of orbit |
| H05.231 | ICD-10 | Hemorrhage of right orbit |
| H05.232 | ICD-10 | Hemorrhage of left orbit |
| H05.233 | ICD-10 | Hemorrhage of bilateral orbit |
| H05.239 | ICD-10 | hemorrhage of unspecified orbit |
| H11.3 | ICD-10 | Conjunctival hemorrhage |
| H11.30 | ICD-10 | Conjunctival hemorrhage, unspecified eye |
| H11.31 | ICD-10 | Conjunctival hemorrhage, right eye |
| H11.32 | ICD-10 | Conjunctival hemorrhage, left eye |
| H11.33 | ICD-10 | conjunctival hemorrhage, bilateral |
| H21.03 | ICD-10 | hyphema, bilateral |
| H31.3 | ICD-10 | Choroidal hemorrhage and rupture |
| H31.30 | ICD-10 | Unspecified choroidal hemorrhage |
| H31.301 | ICD-10 | Unspecified choroidal hemorrhage, right eye |
| H31.302 | ICD-10 | Unspecified choroidal hemorrhage, left eye |
| H31.303 | ICD-10 | Unspecified choroidal hemorrhage, bilateral |
| H31.309 | ICD-10 | unspecified choroidal hemorrhage, unspecified eye |
| H31.31 | ICD-10 | Expulsive choroidal hemorrhage |
| H31.311 | ICD-10 | Expulsive choroidal hemorrhage, right eye |
| H31.312 | ICD-10 | Expulsive choroidal hemorrhage, left eye |
| H31.313 | ICD-10 | Expulsive choroidal hemorrhage, bilateral |
| H31.319 | ICD-10 | expulsive choroidal hemorrhage, unspecified eye |
| H31.32 | ICD-10 | Choroidal rupture |
| H31.41 | ICD-10 | Hemorrhagic choroidal detachment |
| H31.411 | ICD-10 | Hemorrhagic choroidal detachment, right eye |
| H31.412 | ICD-10 | Hemorrhagic choroidal detachment, left eye |
| H31.413 | ICD-10 | Hemorrhagic choroidal detachment, bilateral |
| H31.419 | ICD-10 | hemorrhagic choroidal detachment, unspecified eye |
| H35.6 | ICD-10 | Retinal hemorrhage |
| H35.60 | ICD-10 | retinal hemorrhage, unspecified eye |
| H35.61 | ICD-10 | Retinal hemorrhage, right eye |
| H35.62 | ICD-10 | Retinal hemorrhage, left eye |
| H35.63 | ICD-10 | Retinal hemorrhage, bilateral |
| H35.73 | ICD-10 | Hemorrhagic detachment of retinal pigment epithelium |
| H35.731 | ICD-10 | Hemorrhagic detachment of retinal pigment epithelium, right eye |
| H35.732 | ICD-10 | Hemorrhagic detachment of retinal pigment epithelium, left eye |
| H35.733 | ICD-10 | Hemorrhagic detachment of retinal pigment epithelium, bilateral |
| H35.739 | ICD-10 | hemorrhagic detachment of retinal pgiment epithelium unspecified eye |
| H43.1 | ICD-10 | Vitreous hemorrhage |
| H43.10 | ICD-10 | Vitreous hemorrhage, unspecified eye |
| H43.11 | ICD-10 | Vitreous hemorrhage, right eye |
| H43.12 | ICD-10 | Vitreous hemorrhage, left eye |
| H43.13 | ICD-10 | vitreous hemorrhage, bilateral |
| H44.819 | ICD-10 | hemophthalmos, unspecified ey |
| H47.02 | ICD-10 | Hemorrhage in optic nerve sheath |
| H47.021 | ICD-10 | Hemorrhage in optic nerve sheath, right eye |
| H47.022 | ICD-10 | Hemorrhage in optic nerve sheath, left eye |
| H47.023 | ICD-10 | Hemorrhage in optic nerve sheath, bilateral |
| H47.029 | ICD-10 | hemorrhage in optic nerve sheath, unspecified eye |
| H59.111 | ICD-10 | intraoperative hemorrhage and hematoma of right eye and adnexa complicating an ophthalmic procedure |
| H59.112 | ICD-10 | intraoperative hemorrhage and hematoma of left eye and adnexa complicating an ophthalmic procedure |
| H59.113 | ICD-10 | intraoperative hemorrhage and hematoma of eye and adnexa complicating an ophthalmic procedure, bilateral |
| H59.119 | ICD-10 | intraoperative hemorrhage and hematoma of unspecified eye and adnexa complicating an ophthalmic procedure |
| H59.121 | ICD-10 | intraoperative hemorrhage and hematoma of right eye and adnexa complicating other procedure |
| H59.122 | ICD-10 | intraoperative hemorrhage and hematoma of left eye and adnexa complicating other procedure |
| H59.123 | ICD-10 | intraoperative hemorrhage and hematoma of eye and adnexa complicating other procedure, bilateral |
| H59.129 | ICD-10 | intraoperative hemorrhage and hematoma of unspecified eye and adnexa complicating other procedure |
| H59.311 | ICD-10 | postprocedural hemorrhage and hematoma of right eye and adnexa following an ophthalmic procedure |
| H59.312 | ICD-10 | postprocedural hemorrhage and hematoma of left eye and adnexa following an ophthalmic procedure |
| H59.313 | ICD-10 | postprocedural hemorrhage and hematoma of eye and adnexa following an ophthalmic procedure, bilateral |
| H59.319 | ICD-10 | postprocedural hemorrhage and hematoma of unspecified eye and adnexa following an ophthalmic procedure |
| H59.321 | ICD-10 | postprocedural hemorrhage and hematoma of right eye and adnexa following other procedure |
| H59.322 | ICD-10 | postprocedural hemorrhage and hematoma of left eye and adnexa following other procedure |
| H59.323 | ICD-10 | postprocedural hemorrhage and hematoma of eye and adnexa following other procedure, bilateral |
| H59.329 | ICD-10 | postprocedural hemorrhage and hematoma of unspecified eye and adnexa following other procedure |
| H95.21 | ICD-10 | intraoperative hemorrhage and hematoma of ear and mastoid process complicating a procedure on the ear and mastoid process |
| H95.22 | ICD-10 | intraoperative hemorrhage and hematoma of ear and mastoid process complicating other procedure |
| H95.41 | ICD-10 | postprocedural hemorrhage and hematoma of ear and mastoid process following a procedure on the ear and mastoid process |
| H95.42 | ICD-10 | postprocedural hemorrhage and hematoma of ear and mastoid process following other procedure |
| I31.2 | ICD-10 | Hemopericardium, not elsewhere classified |
| I60 | ICD-10 | Nontraumatic subarachnoid hemorrhage |
| I60.0 | ICD-10 | Nontraumatic subarachnoid hemorrhage from carotid siphon and bifurcation |
| I60.00 | ICD-10 | Nontraumatic subarachnoid hemorrhage from unspecified carotid siphon and bifurcation |
| I60.01 | ICD-10 | Nontraumatic subarachnoid hemorrhage from right carotid siphon and bifurcation |
| I60.02 | ICD-10 | Nontraumatic subarachnoid hemorrhage from left carotid siphon and bifurcation |
| I60.1 | ICD-10 | Nontraumatic subarachnoid hemorrhage from middle cerebral artery |
| I60.10 | ICD-10 | Nontraumatic subarachnoid hemorrhage from unspecified middle cerebral artery |
| I60.11 | ICD-10 | Nontraumatic subarachnoid hemorrhage from right middle cerebral artery |
| I60.12 | ICD-10 | Nontraumatic subarachnoid hemorrhage from left middle cerebral artery |
| I60.2 | ICD-10 | Nontraumatic subarachnoid hemorrhage from anterior communicating artery |
| I60.3 | ICD-10 | Nontraumatic subarachnoid hemorrhage from posterior communicating artery |
| I60.30 | ICD-10 | Nontraumatic subarachnoid hemorrhage from unspecified posterior communicating artery |
| I60.31 | ICD-10 | Nontraumatic subarachnoid hemorrhage from right posterior communicating artery |
| I60.32 | ICD-10 | Nontraumatic subarachnoid hemorrhage from left posterior communicating artery |
| I60.4 | ICD-10 | Nontraumatic subarachnoid hemorrhage from basilar artery |
| I60.5 | ICD-10 | Nontraumatic subarachnoid hemorrhage from vertebral artery |
| I60.50 | ICD-10 | Nontraumatic subarachnoid hemorrhage from unspecified vertebral artery |
| I60.51 | ICD-10 | Nontraumatic subarachnoid hemorrhage from right vertebral artery |
| I60.52 | ICD-10 | Nontraumatic subarachnoid hemorrhage from left vertebral artery |
| I60.6 | ICD-10 | Nontraumatic subarachnoid hemorrhage from other intracranial arteries |
| I60.7 | ICD-10 | Nontraumatic subarachnoid hemorrhage from unspecified intracranial artery |
| I60.8 | ICD-10 | Other nontraumatic subarachnoid hemorrhage |
| I60.9 | ICD-10 | nontraumatic subarachnoid hemorrhage, unspecified |
| I61 | ICD-10 | Nontraumatic intracerebral hemorrhage |
| I61.0 | ICD-10 | Nontraumatic intracerebral hemorrhage in hemisphere, subcortical |
| I61.1 | ICD-10 | Nontraumatic intracerebral hemorrhage in hemisphere, cortical |
| I61.2 | ICD-10 | Nontraumatic intracerebral hemorrhage in hemisphere, unspecified |
| I61.3 | ICD-10 | Nontraumatic intracerebral hemorrhage in brain stem |
| I61.4 | ICD-10 | Nontraumatic intracerebral hemorrhage in cerebellum |
| I61.5 | ICD-10 | Nontraumatic intracerebral hemorrhage, intraventricular |
| I61.6 | ICD-10 | Nontraumatic intracerebral hemorrhage, multiple localized |
| I61.8 | ICD-10 | Other nontraumatic intracerebral hemorrhage |
| I61.9 | ICD-10 | nontraumatic intracerebral hemorrhage, unspecified |
| I62 | ICD-10 | Other and unspecified nontraumatic intracranial hemorrhage |
| I62.0 | ICD-10 | Nontraumatic subdural hemorrhage |
| I62.00 | ICD-10 | nontraumatic subdural hemorrhage, unspecified |
| I62.01 | ICD-10 | Nontraumatic acute subdural hemorrhage |
| I62.02 | ICD-10 | Nontraumatic subacute subdural hemorrhage |
| I62.03 | ICD-10 | Nontraumatic chronic subdural hemorrhage |
| I62.1 | ICD-10 | nontraumatic extradural hemorrhage |
| I62.9 | ICD-10 | nontraumatic intracranial hemorrhage, unspecified |
| I85.01 | ICD-10 | esophageal varices with bleeding |
| I85.11 | ICD-10 | secondary esophageal varices with bleeding |
| I97.410 | ICD-10 | intraoperative hemorrhage and hematoma of a circulatory system organ or structure complicating a cardiac catheterization |
| I97.411 | ICD-10 | intraoperative hemorrhage and hematoma of a circulatory system organ or structure complicating a cardiac bypass |
| I97.418 | ICD-10 | intraoperative hemorrhage and hematoma of a circulatory system organ or structure complicating other circulatory system procedure |
| I97.42 | ICD-10 | intraoperative hemorrhage and hematoma of a circulatory system organ or structure complicating other procedure |
| I97.610 | ICD-10 | postprocedural hemorrhage and hematoma of a circulatory system organ or structure following a cardiac catheterization |
| I97.611 | ICD-10 | postprocedural hemorrhage and hematoma of a circulatory system organ or structure following cardiac bypass |
| I97.618 | ICD-10 | postprocedural hemorrhage and hematoma of a circulatory system organ or structure following other circulatory system procedure |
| I97.62 | ICD-10 | postprocedural hemorrhage and hematoma of a circulatory system organ or structure following other procedure |
| J95.61 | ICD-10 | intraoperative hemorrhage and hematoma of a respiratory system organ or structure complicating a respiratory system procedure |
| J95.62 | ICD-10 | intraoperative hemorrhage and hematoma of a respiratory system organ or structure complicating other procedure |
| J95.830 | ICD-10 | postprocedural hemorrhage and hematoma of a respiratory system organ or structure following a respiratory system procedure |
| J95.831 | ICD-10 | postprocedural hemorrhage and hematoma of a respiratory system organ or structure following other procedure |
| K22.11 | ICD-10 | ulcer of esophagus with bleeding |
| K22.6 | ICD-10 | gastro-esophageal laceration-hemorrhage syndrome |
| K22.8 | ICD-10 | other specified diseases of esophagus |
| K25.0 | ICD-10 | acute gastric ulcer with hemorrhage |
| K25.2 | ICD-10 | acute gastric ulcer with both hemorrhage and perforation |
| K25.4 | ICD-10 | chronic or unspecified gastric ucler with hemorrhage |
| K25.6 | ICD-10 | chronic or unspecified gastric ulcer with both hemorrhage and perforation |
| K26.0 | ICD-10 | acute duodenal ulcer with hemorrhage |
| K26.2 | ICD-10 | acute duodenal ulcer with both hemorrhage and perforation |
| K26.4 | ICD-10 | chronic or unspecified duodenal ulcer with hemorrhage |
| K26.6 | ICD-10 | chronic or unspecified duodenal ulcer with both hemorrhage and perforation |
| K27.0 | ICD-10 | acute peptic ulcer, site, unspecified, with hemorrhage |
| K27.2 | ICD-10 | acute peptic ulcer, site unspecified, with both hemorrhage and perforation |
| K27.4 | ICD-10 | chronic or unspecified peptic ulcer, site unspecified, with hemorrhage |
| K27.6 | ICD-10 | chronic or unspecified peptic ulcer, site unspecified, with both hemorrhage and perforation |
| K28.0 | ICD-10 | acute gastrojejunal ulcer with hemorrhage |
| K28.2 | ICD-10 | acute gastrojejunal ulcer with both hemorrhage and perforation |
| K28.4 | ICD-10 | chronic or unspecified gastrojejunal ulcer with hemorrhage |
| K28.6 | ICD-10 | chronic or unspecified gastrojejunal ulcer with both hemorrhage and perforation |
| K29.01 | ICD-10 | acute gastritis with bleeding |
| K29.21 | ICD-10 | alcoholic gastritis with bleeding |
| K29.41 | ICD-10 | chronic atrophic gastritis with bleeding |
| K29.51 | ICD-10 | unspecified chronic gastritis with bleeding |
| K29.61 | ICD-10 | other gastritis with bleeding |
| K29.71 | ICD-10 | gastritis, unspecified, with bleeding |
| K29.81 | ICD-10 | Duodentis with bleeding |
| K29.91 | ICD-10 | gastroduodenitis, unspecified with bleeding |
| K31.811 | ICD-10 | angiodysplasia of stomach and duodenum with bleeding |
| K31.82 | ICD-10 | dieulafoy lesion (hemorrhagic) of stomach and duodenum |
| K55.21 | ICD-10 | angiodysplasia of colon with hemorrhage |
| K57.11 | ICD-10 | diverticulosis of small intestine without performation or abscess with bleeding |
| K57.13 | ICD-10 | diverticulitis of small intestine without perforation or abscess with bleeding |
| K57.31 | ICD-10 | diverticulosis of large intestine without perforation or abscess with bleeding |
| K57.33 | ICD-10 | diverticulitis of large intestine without perforation or abscess with bleeding |
| K62.5 | ICD-10 | hemorrhage of anus and rectum |
| K63.81 | ICD-10 | dieulafoy lesion of intestine |
| K64.4 | ICD-10 | residual hemorrhoidal skin tags |
| K64.8 | ICD-10 | Other hemorrhoids |
| K66.1 | ICD-10 | hemoperitoneum |
| K91.61 | ICD-10 | intraoperative hemorrhage and hematoma of a digestive system organ or structure complicating a digestive sytem procedure |
| K91.62 | ICD-10 | intraoperative hemorrhage and hematoma of a digestive system organ or structure complicating other procedure |
| K91.840 | ICD-10 | postprocedural hemorrhage and hematoma of a digestive system organ or structure following a digestive system procedure |
| K91.841 | ICD-10 | postprocedural hemorrhage and hematoma of a digestive system organ or structure following other procedure |
| K92.0 | ICD-10 | Hematemesis |
| K92.1 | ICD-10 | Melena |
| K92.2 | ICD-10 | Gastrointestinal hemorrhage, unspecified |
| L76.01 | ICD-10 | intraoperative hemorrhage and hematoma of skin and subcutaneous tissue complicating a dermatologic procedure |
| L76.02 | ICD-10 | intraoperative hemorrhage and hematoma of skin and subcutaneous tissue complicating other procedure |
| L76.21 | ICD-10 | postprocedural hemorrhage and hematoma of skin and subcutaneous tissue following a dermatologic procedure |
| L76.22 | ICD-10 | postprocedural hemorrhage and hematoma of skin and subcutaneous tissue following other procedure |
| M25.00 | ICD-10 | hemarthrosis, unspecified joint |
| M25.019 | ICD-10 | hemarthrosis, unspecified shoulder |
| M25.029 | ICD-10 | hemarthrosis, unspecified elbow |
| M25.039 | ICD-10 | hemarthrosis, unspecified wrist |
| M25.049 | ICD-10 | hemarthrosis, unspecified hand |
| M25.059 | ICD-10 | hemarthrosis, unspecified hip |
| M25.069 | ICD-10 | hemarthrosis, unspecified knee |
| M25.073 | ICD-10 | hemarthrosis, unspecified ankle |
| M25.076 | ICD-10 | hemarthrosis, unsepcified foot |
| M25.08 | ICD-10 | hemarthrosis, unspecified vertebrae |
| M96.810 | ICD-10 | intraoperative hemorrhage and hematoma of a musculoskeletal structure complicating a musculoskeletal system procedure |
| M96.811 | ICD-10 | intraoperative hemorrhage and hematoma of a musculoskeletal structure complicating other procedure |
| M96.830 | ICD-10 | postprocedural hemorrhage and hematoma of a musculoskeletal structure following a musculoskeletal system procedure |
| M96.831 | ICD-10 | postprocedural hemorrhage and hematoma of a musculoskeletal structure following other procedure |
| N99.61 | ICD-10 | intraoperative hemorrhage and hematoma of a genitourinary system organ or structure complicating a genitourinary system procedure |
| N99.62 | ICD-10 | intraoperative hemorrhage and hematoma of a genitourinary system organ or structure complicating other procedure |
| N99.820 | ICD-10 | postprocedural hemorrhage and hematoma of a genitourinary system organ or structure following a genitourinary system procedure |
| N99.821 | ICD-10 | postprocedural hemorrhage and hematoma of a genitourinary system organ or structure following other procedure |
| R04.0 | ICD-10 | epistaxis |
| R04.1 | ICD-10 | hemorrhage from throat |
| R04.2 | ICD-10 | hemoptysis |
| R04.9 | ICD-10 | hemorrhage from respiratory passages, unspecified |
| R31.0 | ICD-10 | gross hematuria |
| R31.9 | ICD-10 | hematuria, unspecified |
| R58 | ICD-10 | hemorrhage, not elsewhere classified |
