# Supplementary material for: Comparing outcomes and costs among warfarin-sensitive patients versus warfarin-insensitive patients using The Right Drug, Right Dose, Right Time: Using genomic data to individualize treatment (RIGHT) 10K warfarin cohort
Source: PLoS One. 2020 May 19;15(5):e0233316. doi: 10.1371/journal.pone.0233316 (PMC7237006; doi:10.1371/journal.pone.0233316)
Supplement: S2 Appendix — This table contains the standard Clinical Classification Software categories used from the Healthcare Cost and Utilization Project to aid in the identification of important risk factors of interest. (DOCX) [file pone.0233316.s002.docx]

**S2 Appendix. CCS categories used to identify risk factors**

| **CCS Category Number** | **CCS Category Description** |
| --- | --- |
| 1.1 | Bacterial Infection |
| 1.2 | Mycoses |
| 1.3.3 | Other viral infections |
| 1.4 | Other infections; including parasitic |
| 2 | Neoplasms |
| 3.1 | Thyroid Disorders |
| 3.2 | Diabetes without complication |
| 3.3 | Diabetes with complications |
| 4.1 | Anemia |
| 4.2 | Coagulation and hemorrhagic disorders |
| 5.4 | Delirium, dementia, and amnestic and other cognitive disorders |
| 5.7-5.15 | Mood Disorders, alcohol related disorders, mental health disorders |
| 7.1 | Hypertension |
| 7.2 | Diseases of the heart |
| 7.3 | Cerebrovascular Disorders |
| 7.3.1.1 | Intracranial hemorrhage |
| 9.4.2 | Gastroduodenal ulcer |
| 9.8 | Liver disease |
| 10.1.1-10.1.3 | Renal failure |
| 10.1.5.1 | Calculus of kidney |
| 10.1.6.1 | Hydronephrosis |
| 10.1.8.1 | Hematuria |
| 11.3.2.3 | Other hemorrhage during pregnancy; childbirth and the puerperium |
| 16.1-16.10 | Trauma |
| 17.1.2 | Fever of unknown origin |
