# Supplementary material for: Comparing outcomes and costs among warfarin-sensitive patients versus warfarin-insensitive patients using The Right Drug, Right Dose, Right Time: Using genomic data to individualize treatment (RIGHT) 10K warfarin cohort
Source: PLoS One. 2020 May 19;15(5):e0233316. doi: 10.1371/journal.pone.0233316 (PMC7237006; doi:10.1371/journal.pone.0233316)
Supplement: S1 Table — These tables include the primary results (Tables 1 through 5) for the subgroup analysis performed on the empaneled patient population. (DOCX) [file pone.0233316.s003.docx]

**S1 Table. Results tables for empaneled subgroup**

**S1A Table: Distribution of Warfarin Related Genetic Variants**

|  | **Frequency** |
| --- | --- |
| **CYP2C9 Genotype** | **N (%)** |
| 1/1 | 354 (62.0%) |
| 1/2 | 119 (20.8%) |
| 1/3 | 64 (11.2%) |
| 2/2 | 8 (1.4%) |
| 2/3 | 11 (1.9%) |
| 3/3 | 1 (0.2%) |
| Other Variant | 14 (2.5%) |
|  |  |
| **Warfarin VKORC1 c.-1639 Genotype** |  |
| A/A | 80 (14.0%) |
| G/A | 279 (48.9%) |
| G/G | 212 (37.1%) |
| Other | 0 (0.0%) |
|  |  |
| **Combined Phenotype** |  |
| Normal Sensitivity to Warfarin | 352 (61.6%) |
| Intermediate Sensitivity to Warfarin | 201 (35.2%) |
| High Sensitivity to Warfarin | 18 (3.2%) |

**S1B Table: Comparison of Baseline Characteristics across Exposure Groups**

| **Baseline Characteristic** | **Exposure Group** | | **Total**  **(N = 571)** | **P-Value** |
| --- | --- | --- | --- | --- |
|  | **Normal**  **(N = 352)** | **Sensitive**  **(N = 219)** |  |  |
| **Age** |  |  |  | 0.43 |
| <60 | 21.1% | 17.0% | 19.5% |  |
| 60-64 | 15.4% | 14.2% | 14.9% |  |
| 65-70 | 20.8% | 27.1% | 23.2% |  |
| 70-74 | 23.9% | 22.0% | 23.2% |  |
| 75+ | 18.8% | 17.0% | 19.5% |  |
| **% Male** | 53.0% | 47.2% | 50.8% | 0.18 |
| **% White** | 98.3% | 99.5% | 98.8% | 0.78 |
| **% Hispanic** | 2.8% | 0.9% | 2.1% | 0.14 |
| **BMI** |  |  |  | 0.57 |
| Underweight | 0.6% | 0.0% | 0.4% |  |
| Normal | 12.8% | 11.4% | 12.3% |  |
| Overweight | 24.4% | 28.8% | 26.1% |  |
| Obese | 47.4% | 43.8% | 46.1% |  |
| Unknown | 14.8% | 16.0% | 15.2% |  |
| **Education Level** |  |  |  | 0.24 |
| Some high school | 1.1% | 0.9% | 1.1% |  |
| High school/GED | 16.0% | 23.4% | 18.8% |  |
| Some college or 2 yr degree | 34.2% | 28.4% | 32.0% |  |
| 4 yr college degree | 15.1% | 15.1% | 15.1% |  |
| Post graduate studies | 33.6% | 32.1% | 33.0% |  |
| **Smoking Status** |  |  |  | 0.15 |
| Non-User | 59.7% | 53.9% | 57.4% |  |
| Current User | 15.3% | 20.5% | 17.3% |  |
| Former User | 21.3% | 19.2% | 20.5% |  |
| Unknown | 2.7% | 6.4% | 4.7% |  |
| **Charlson Index** |  |  |  | 0.03 |
| 0 | 40.9% | 32.0% | 37.5% |  |
| 1 | 24.1% | 20.1% | 22.6% |  |
| 2 | 13.4% | 16.0% | 14.4% |  |
| 3 | 8.0% | 13.2% | 10.0% |  |
| 4 or more | 13.6% | 18.7% | 15.6% |  |
| **% with any prior bleed** | 27.0% | 29.2% | 28.9% | 0.56 |
| **% received anticoagulant in year prior** | 9.4% | 11.0% | 10.0% | 0.54 |
| **Hypertension** | 56.3% | 57.5% | 56.7% | 0.76 |
| **Cardiovascular Disease** | 65.6% | 67.1% | 66.2% | 0.71 |
| **Myocardial Infarction** | 5.4% | 9.6% | 7.0% | 0.06 |
| **Cerebrovascular Disease** | 9.1% | 11.4% | 10.0% | 0.37 |
| **Anemia** | 22.7% | 22.4% | 22.6% | 0.92 |
| **Diabetes** | 33.0% | 37.0% | 34.5% | 0.32 |
| **Malignancy** | 34.1% | 40.6% | 36.6% | 0.11 |
| **Liver disease** | 6.8% | 8.2% | 7.4% | 0.53 |
| **Diseases of the Urinary System** | 28.7% | 29.2% | 28.9% | 0.89 |
| **Thyroid Disorders** | 13.1% | 16.9% | 14.5% | 0.21 |
| **Mental Illness** | 28.4% | 31.5% | 29.6% | 0.43 |
| **Coagulation and Hemorrhagic Disorders** | 7.4% | 7.8% | 7.5% | 0.87 |

**S1C Table: Summary of Outcomes across Exposure Groups**

|  | **Normal**  **(N = 722)** | | **Sensitive**  **(N = 421)** | | **P-Value** |
| --- | --- | --- | --- | --- | --- |
|  | Mean (SD) | Median (Q1,Q3) | Mean (SD) | Median (Q1,Q3) |  |
| **Bleeding Events Among Those With Bleeds (#/patient)** | 3.44 (2.78) | 2 (1,5) | 4.5 (1.5,5) | 3 (2, 5.5) | 0.41 |
| **All-Cause Costs ($)** |  |  |  |  |  |
| Total Costs | $16,174.55 ($23,181.03) | $8,327.61  ($3,942.22, $20,291.72) | $17,167.67 ($24,459.66) | $10,448.62 ($3,960.36, $19,934.54) | 0.33 |
| Inpatient Costs | $9,676.49 ($20,287.21) | $519.83 ($0.00, $14,075.85) | $10,196.18 ($18,966.48) | $2,016.92 ($0.00, $15,024.68) | 0.15 |
| Emergency Department Costs | $293.34 ($913.57) | $0.00  ($0.00, $0.00) | $272.35 ($837.17) | $0.00  ($0.00, $0.00) | 0.99 |
| Hospital Outpatient Costs | $3,109.13  ($6,715.62) | $758.52  ($0.00, $3,411.27) | $3,443.64 ($8,534.53) | $886.80 ($0.00, $3,590.23) | 0.69 |
| Clinic Costs | $3,095.60 ($3,628.80) | $2,331.83  ($1,308.85, $3,852.03) | $3,255.51 ($3,966.66) | $2,430.75 ($1,337.73, $4,037.46) | 0.65 |
| **CV-Related Costs ($)** |  |  |  |  |  |
| Total Costs | $5,782.67 ($17,530.47) | $842.16  ($103.30, $3,430.05) | $3,873.20 ($8,373.67) | $878.59  ($82.19, $3,649.23) | 0.99 |
| Inpatient Costs | $3,538.02 ($1,556.78) | $0.00  ($0.00, $150.45) | $1,957.80 ($6,897.05) | $0.00  ($0.00, $231.04) | 0.60 |
| Emergency Department Costs | $113.52 ($546.98) | $0.00  ($0.00, $0.00) | $80.40 ($403.17) | $0.00  ($0.00, $0.00) | 0.60 |
| Hospital Outpatient Costs | $1,399.60 ($3,810.63) | $0.00  ($0.00, $758.79) | $1,121.14 ($3,147.33) | $0.00  ($0.00, $512.96) | 0.50 |
| Clinic Costs | $731.53 ($927.42) | $393.61  ($49.88, $1,010.67) | $713.86 ($867.57) | $421.46  ($74.79, $989.09) | 0.93 |

**S1D Table: Predicted probabilities of experiencing a major bleeding event and predicted average number of bleeding events across exposure groups for warfarin sensitivity^a^**

|  | **Regression Estimates (odds ratio and incident rate ratios)** | **Predictive Margins (predicted probabilities of predicted average number of events)** | |  |
| --- | --- | --- | --- | --- |
| **Outcome** | **Sensitive vs. Normal** | **Normal** | **Sensitive** | **P-Value** |
| **Experiencing a Major Bleeding Event** |  |  |  | 0.83 |
| Estimate | 0.9383 | 0.0997 | 0.0936 |  |
| 95% CI | (0.5182, 1.6992) | (0.0687, 0.1448) | (0.0580, 0.1509) |  |
| **Number of Major Bleeding Events (entire study sample)** |  |  |  | 0.64 |
| Estimate | 1.2255 | 0.2361 | 0.2894 |  |
| 95% CI | (0.5265, 2.8524) | (0.1148, 0.3575) | (0.1022, 0.4766) |  |
| **Number of Major Bleeding Events (for those who experienced a bleed)** |  |  |  | 0.50 |
| Estimate | 1.1347 | 3.234 | 3.6696 |  |
| 95% CI | (0.7838, 1.6426) | (2.4725, 3.9956) | (2.6243, 4.7149) |  |

^a^ Each estimated was generated from a multivariate regression model regressing warfarin sensitivity on each of the bleeding outcomes, controlling for the following adjusting variables: age, Charlson index, history of myocardial infarction, history of diabetes, and history of cancer

**S1E Table: Predicted probabilities of incurring costs and predicted average costs across exposure groups for warfarin sensitivity^a^**

|  | Predicted Probabilities of Incurring Costs (Logit Model^b^)  Mean (95% CI) | | Predicted Mean Costs (Gamma Model^c^)  Mean (95% CI) | | Combined – Predicted Mean Costs (Two-Part Model)  Mean (95% CI) | |
| --- | --- | --- | --- | --- | --- | --- |
| Outcome | Normal | Sensitive to Warfarin | Normal | Sensitive to Warfarin | Normal | Sensitive to Warfarin |
| **All Cause** |  |  |  |  |  |  |
| Total Costs |  |  | 16744  (14463.99, 19024.01) | 16510.46  (13790.37, 19230.56) |  |  |
| Inpatient Costs | 0.666  (0.6158, 0.7162) | 0.7054  (0.6438, 0.7669) | 14959.48  (12302.35, 17616.61) | 14159.39  (11129.28, 17189.51) | 9900.19  (7995.96, 11804.42) | 9905.95  (7627.40, 12184.50) |
| Emergency Department Costs | 0.1751  (0.1347, 0.2155) | 0.1735  (0.1225, 0.2245) | 1626.46  (1271.77, 1981.16) | 1499.35  (1082.96, 1915.75) | 296.48  (202.38, 390.58) | 270.83  (163.59, 378.07) |
| Hospital Outpatient Costs | 0.6413  (0.5888, 0.6938) | 0.6417  (0.5757, 0.7078) | 4994.43  (3976.12, 6012.75) | 5351.15  (3997.46, 6704.84) | 3144.39  (2458.73, 3830.06) | 3371.03  (2455.47, 4286.58) |
| Clinic Costs |  |  | 3201.35  (2888.96, 3513.74) | 3118.92  (2754.59, 3483.24) |  |  |
|  |  |  |  |  |  |  |
| **CV-Related** |  |  |  |  |  |  |
| Total Costs | 0.8448  (0.8029, 0.8868) | 0.8231  (0.7677, 0.8785) | 7255.25*  (5191.07, 9319.43) | 4676.83*  (3083.07, 6270.59) | 5187.78*  (3781.99, 6593.57) | 3254.69*  (2117.76, 4391.63) |
| Inpatient Costs | 0.2799  (0.2309, 0.3290) | 0.2757  (0.2142, 0.3373) | 12692.28  (7405.79, 17978.77) | 6850.12  (3502.39, 10197.84) | 3704.15  (2040.58, 5367.72) | 1973.81  (930.98, 3016.64) |
| Emergency Department Costs | 0.0544  (0.0299, 0.0788) | 0.0506  (0.0214, 0.0798) | 1835.96  (1320.04, 2351.88) | 1322.86  (835.46, 1810.25) | 116.45  (56.99, 175.91) | 78.29  (25.74, 130.84) |
| Hospital Outpatient Costs | 0.3938  (0.3417, 0.4459) | 0.3542  (0.2894, 0.4191) | 3466.44  (2523.71, 4409.17) | 3276.45  (2038.67, 4514.23) | 1375.46  (961.17, 1789.74) | 1175.86  (684.69, 1667.04) |
| Clinic Costs | 0.8383  (0.7957, 0.8809) | 0.8407  (0.7892, 0.8921) | 921.72  (810.55, 1032.89) | 857.90  (730.29, 985.52) | 746.36  (649.23, 843.49) | 696.84  (583.87, 809.82) |

^a^ Each estimated was generated from a multivariate regression model regressing warfarin sensitivity on each of the cost categories, controlling for the following adjusting variables: age, charlson index, history of myocardial infarction, history of diabetes, and history of cancer

^b^ Rows that have estimates populated for the Logit model were run using a two-part model.

^c^ Rows that only have estimates for the Gamma model were run with a regular generalized linear model using a gamma distribution to model costs.

* represents significance at the <0.05 level

** represents significance at the <0.01 level

** represents significance at the <0.001 level
