# Supplementary material for: Comparing outcomes and costs among warfarin-sensitive patients versus warfarin-insensitive patients using The Right Drug, Right Dose, Right Time: Using genomic data to individualize treatment (RIGHT) 10K warfarin cohort
Source: PLoS One. 2020 May 19;15(5):e0233316. doi: 10.1371/journal.pone.0233316 (PMC7237006; doi:10.1371/journal.pone.0233316)
Supplement: S2 Table — These tables contain the full regression results (parameter estimates, standard errors, 95% confidence intervals, and p-values for all covariates included in the multivariate models), with models being performed on the full study population. A separate table for each regression outcome of interest is included. (DOCX) [file pone.0233316.s004.docx]

**S2 Table. Complete regression results for the full study population**

**Model for Experience a Major Bleeding Event**

|  | Odds Ratio | Std. Err. | P>z | [95% Conf. | Interval] |
| --- | --- | --- | --- | --- | --- |
|  |  |  |  |  |  |
| 1.warf_sensitivity | 0.841 | 0.190 | 0.443 | 0.541 | 1.308 |
| age |  |  |  |  |  |
| 60-64 years | 0.991 | 0.367 | 0.98 | 0.479 | 2.047 |
| 65-70 years | 0.905 | 0.303 | 0.766 | 0.470 | 1.743 |
| 70-74 years | 0.890 | 0.308 | 0.735 | 0.452 | 1.752 |
| 75+ years | 1.942 | 0.638 | 0.044 | 1.020 | 3.698 |
| ch_index | 1.123 | 0.052 | 0.013 | 1.025 | 1.230 |
| 1.dx1 | 1.511 | 0.558 | 0.263 | 0.733 | 3.115 |
| 1.diab_met | 1.348 | 0.315 | 0.201 | 0.853 | 2.131 |
| 1.malig | 1.290 | 0.295 | 0.265 | 0.824 | 2.019 |

**Model for Number of Major Bleeding Events (entire study sample)**

|  | IRR | Std. Err. | P>z | [95% Conf. | Interval] |
| --- | --- | --- | --- | --- | --- |
|  |  |  |  |  |  |
| 1.warf_sensitivity | 0.898 | 0.291 | 0.74 | 0.475 | 1.696 |
| age |  |  |  |  |  |
| 60-64 years | 0.799 | 0.401 | 0.655 | 0.299 | 2.137 |
| 65-70 years | 1.009 | 0.454 | 0.984 | 0.418 | 2.436 |
| 70-74 years | 0.903 | 0.423 | 0.828 | 0.360 | 2.264 |
| 75+ years | 1.492 | 0.777 | 0.442 | 0.538 | 4.139 |
| ch_index | 1.206 | 0.104 | 0.03 | 1.019 | 1.429 |
| 1.dx1 | 2.581 | 1.593 | 0.124 | 0.770 | 8.654 |
| 1.diab_met | 1.136 | 0.407 | 0.722 | 0.562 | 2.295 |
| 1.malig | 1.815 | 0.602 | 0.072 | 0.947 | 3.478 |

Pseudo R2 0.0184

**Model for Number of Major Bleeding Events (for those who experienced a bleed)**

|  | IRR | Std. Err. | P>z | [95% Conf. | Interval] |
| --- | --- | --- | --- | --- | --- |
|  |  |  |  |  |  |
| 1.warf_sensitivity | 1.136 | 0.182 | 0.426 | 0.830 | 1.555 |
| age |  |  |  |  |  |
| 60-64 years | 0.962 | 0.270 | 0.89 | 0.555 | 1.668 |
| 65-70 years | 1.032 | 0.258 | 0.899 | 0.633 | 1.683 |
| 70-74 years | 1.052 | 0.266 | 0.841 | 0.641 | 1.727 |
| 75+ years | 0.922 | 0.230 | 0.746 | 0.565 | 1.505 |
| ch_index | 1.066 | 0.030 | 0.024 | 1.008 | 1.127 |
| 1.dx1 | 1.303 | 0.315 | 0.274 | 0.811 | 2.092 |
| 1.diab_met | 0.951 | 0.166 | 0.773 | 0.675 | 1.339 |
| 1.malig | 1.352 | 0.219 | 0.063 | 0.984 | 1.856 |

Pseudo R2 0.03

**Model for Total All-Cause Costs**

|  | Coef. | Std. Err. | P>z | [95% Conf. | Interval] |
| --- | --- | --- | --- | --- | --- |
|  |  |  |  |  |  |
| 1.warf_sensitivity | -0.072 | 0.088 | 0.409 | -0.245 | 0.100 |
| age |  |  |  |  |  |
| 60-64 years | -0.162 | 0.137 | 0.235 | -0.430 | 0.106 |
| 65-70 years | -0.190 | 0.123 | 0.121 | -0.431 | 0.050 |
| 70-74 years | -0.314 | 0.129 | 0.015 | -0.567 | -0.062 |
| 75+ years | -0.129 | 0.142 | 0.362 | -0.408 | 0.149 |
| ch_index | 0.133 | 0.023 | 0.000 | 0.088 | 0.177 |
| 1.dx1 | 0.316 | 0.183 | 0.084 | -0.043 | 0.675 |
| 1.diab_met | -0.105 | 0.098 | 0.281 | -0.297 | 0.086 |
| 1.malig | 0.100 | 0.092 | 0.276 | -0.080 | 0.281 |

Log likelihood -12200.15468

AIC 21.42132

BIC -6060.746

**Model for Inpatient All-Cause Costs**

|  | Logit |  |  |  |  | GLM |  |  |  |  |
| --- | --- | --- | --- | --- | --- | --- | --- | --- | --- | --- |
|  | Odds Ratio | Std. Err. | P>z | [95% Conf. | Interval] | Coef. | Std. Err. | P>z | [95% Conf. | Interval] |
|  |  |  |  |  |  |  |  |  |  |  |
| 1.warf_sensitivity | 1.015 | 0.135 | 0.912 | 0.782 | 1.317 | -0.099 | 0.124 | 0.424 | -0.341 | 0.144 |
| age |  |  |  |  |  |  |  |  |  |  |
| 60-64 years | 0.836 | 0.172 | 0.384 | 0.558 | 1.252 | -0.144 | 0.192 | 0.453 | -0.521 | 0.233 |
| 65-70 years | 0.740 | 0.137 | 0.103 | 0.515 | 1.063 | -0.151 | 0.174 | 0.384 | -0.491 | 0.189 |
| 70-74 years | 0.702 | 0.136 | 0.068 | 0.481 | 1.026 | -0.336 | 0.184 | 0.068 | -0.696 | 0.025 |
| 75+ years | 0.766 | 0.167 | 0.220 | 0.500 | 1.173 | -0.114 | 0.199 | 0.567 | -0.504 | 0.276 |
| ch_index | 1.140 | 0.043 | 0.001 | 1.058 | 1.228 | 0.092 | 0.030 | 0.002 | 0.034 | 0.151 |
| 1.dx1 | 2.053 | 0.686 | 0.031 | 1.067 | 3.953 | 0.275 | 0.234 | 0.241 | -0.184 | 0.733 |
| 1.diab_met | 1.320 | 0.199 | 0.065 | 0.982 | 1.775 | -0.147 | 0.138 | 0.286 | -0.417 | 0.123 |
| 1.malig | 0.958 | 0.134 | 0.757 | 0.727 | 1.260 | 0.117 | 0.131 | 0.372 | -0.140 | 0.375 |

Pseudo R2 0.0239

GOF p-value 0.0649

Log likelihood -7991.220654

AIC 21.36508

BIC -3054.851

**Model for ED All-Cause Costs**

|  | Odds Ratio | Std. Err. | P>z | [95% Conf. | Interval] | Coef. | Std. Err. | P>z | [95% Conf. | Interval] |
| --- | --- | --- | --- | --- | --- | --- | --- | --- | --- | --- |
|  |  |  |  |  |  |  |  |  |  |  |
| 1.warf_sensitivity | 0.889 | 0.152 | 0.489 | 0.636 | 1.241 | -0.117 | 0.135 | 0.387 | -0.381 | 0.148 |
| age |  |  |  |  |  |  |  |  |  |  |
| 60-64 years | 0.434 | 0.119 | 0.002 | 0.253 | 0.744 | -0.379 | 0.219 | 0.084 | -0.808 | 0.051 |
| 65-70 years | 0.595 | 0.133 | 0.020 | 0.384 | 0.922 | -0.003 | 0.174 | 0.985 | -0.345 | 0.338 |
| 70-74 years | 0.469 | 0.115 | 0.002 | 0.290 | 0.760 | -0.363 | 0.198 | 0.067 | -0.751 | 0.025 |
| 75+ years | 0.638 | 0.166 | 0.084 | 0.384 | 1.062 | -0.378 | 0.200 | 0.058 | -0.770 | 0.013 |
| ch_index | 1.053 | 0.043 | 0.205 | 0.972 | 1.141 | 0.068 | 0.032 | 0.035 | 0.005 | 0.131 |
| 1.dx1 | 1.870 | 0.564 | 0.038 | 1.036 | 3.377 | -0.396 | 0.225 | 0.078 | -0.837 | 0.045 |
| 1.diab_met | 1.321 | 0.241 | 0.127 | 0.924 | 1.889 | -0.024 | 0.140 | 0.866 | -0.299 | 0.251 |
| 1.malig | 0.884 | 0.160 | 0.497 | 0.621 | 1.260 | 0.058 | 0.142 | 0.685 | -0.221 | 0.336 |

Pseudo R2 0.0234

GOF p-value 0.3713

Log likelihood -1567.559954

AIC 16.60589

BIC -826.9833

**Model for Hospital Outpatient All-Cause Costs**

|  | Logit |  |  |  |  | GLM |  |  |  |
| --- | --- | --- | --- | --- | --- | --- | --- | --- | --- |
|  | Odds Ratio | Std. Err. | P>z | [95% Conf. | Interval] | Coef. | Std. Err. | z | P>z |
|  |  |  |  |  |  |  |  |  |  |
| 1.warf_sensitivity | 1.040 | 0.136 | 0.763 | 0.805 | 1.344 | 0.010 | 0.117 | 0.090 | 0.930 |
| age |  |  |  |  |  |  |  |  |  |
| 60-64 years | 0.768 | 0.152 | 0.180 | 0.521 | 1.130 | -0.054 | 0.185 | -0.290 | 0.771 |
| 65-70 years | 0.990 | 0.178 | 0.956 | 0.697 | 1.408 | -0.180 | 0.159 | -1.130 | 0.258 |
| 70-74 years | 1.096 | 0.208 | 0.628 | 0.756 | 1.590 | -0.066 | 0.162 | -0.410 | 0.684 |
| 75+ years | 1.179 | 0.253 | 0.444 | 0.774 | 1.796 | 0.065 | 0.178 | 0.370 | 0.715 |
| ch_index | 1.218 | 0.048 | 0.000 | 1.126 | 1.316 | 0.116 | 0.027 | 4.310 | 0.000 |
| 1.dx1 | 1.063 | 0.310 | 0.835 | 0.600 | 1.882 | 0.202 | 0.218 | 0.920 | 0.356 |
| 1.diab_met | 1.009 | 0.148 | 0.951 | 0.757 | 1.346 | -0.334 | 0.125 | -2.680 | 0.007 |
| 1.malig | 1.043 | 0.144 | 0.763 | 0.795 | 1.367 | -0.055 | 0.116 | -0.470 | 0.635 |

Pseudo R2 0.0299

GOF p-value 0.2265

Log likelihood -6731.954575

AIC 19.09902

BIC -3528.936

**Model for Clinic All-Cause Costs**

|  | Coef. | Std. Err. | P>z | [95% Conf. | Interval] |
| --- | --- | --- | --- | --- | --- |
|  |  |  |  |  |  |
| 1.warf_sensitivity | -0.012 | 0.054 | 0.829 | -0.118 | 0.094 |
| age |  |  |  |  |  |
| 60-64 years | -0.094 | 0.084 | 0.267 | -0.259 | 0.072 |
| 65-70 years | -0.134 | 0.076 | 0.077 | -0.282 | 0.015 |
| 70-74 years | -0.336 | 0.079 | 0.000 | -0.492 | -0.181 |
| 75+ years | -0.364 | 0.088 | 0.000 | -0.537 | -0.192 |
| ch_index | 0.122 | 0.014 | 0.000 | 0.095 | 0.150 |
| 1.dx1 | -0.146 | 0.113 | 0.194 | -0.367 | 0.074 |
| 1.diab_met | -0.033 | 0.059 | 0.576 | -0.149 | 0.083 |
| 1.malig | 0.165 | 0.057 | 0.004 | 0.053 | 0.277 |

Log likelihood -10216.5256

AIC 17.94127

BIC -6805.675

**Model for Total CV-Related Costs**

|  | Odds Ratio | Std. Err. | P>z | [95% Conf. | Interval] | Coef. | Std. Err. | P>z | [95% Conf. | Interval] |
| --- | --- | --- | --- | --- | --- | --- | --- | --- | --- | --- |
|  |  |  |  |  |  |  |  |  |  |  |
| 1.warf_sensitivity | 1.036 | 0.165 | 0.823 | 0.758 | 1.416 | -0.401 | 0.192 | 0.036 | -0.777 | -0.025 |
| age |  |  |  |  |  |  |  |  |  |  |
| 60-64 years | 0.918 | 0.202 | 0.698 | 0.596 | 1.414 | 0.001 | 0.301 | 0.997 | -0.589 | 0.592 |
| 65-70 years | 1.561 | 0.335 | 0.038 | 1.024 | 2.378 | 0.098 | 0.265 | 0.711 | -0.421 | 0.617 |
| 70-74 years | 1.658 | 0.382 | 0.028 | 1.055 | 2.605 | -0.248 | 0.271 | 0.362 | -0.780 | 0.284 |
| 75+ years | 1.582 | 0.415 | 0.080 | 0.946 | 2.645 | -0.093 | 0.299 | 0.757 | -0.679 | 0.493 |
| ch_index | 1.294 | 0.073 | 0.000 | 1.159 | 1.446 | 0.160 | 0.048 | 0.001 | 0.065 | 0.255 |
| 1.dx1 | 5.045 | 3.687 | 0.027 | 1.204 | 21.135 | 0.483 | 0.353 | 0.171 | -0.209 | 1.174 |
| 1.diab_met | 1.017 | 0.187 | 0.925 | 0.709 | 1.460 | -0.184 | 0.205 | 0.371 | -0.586 | 0.219 |
| 1.malig | 0.991 | 0.168 | 0.958 | 0.711 | 1.381 | -0.014 | 0.195 | 0.944 | -0.396 | 0.369 |

Pseudo R2 0.0574

GOF p-value 0.6673

Log likelihood -8782.197014

AIC 19.45176

BIC -3134.927

**Model for Inpatient CV-Related Costs**

|  | Logit |  |  |  |  | GLM |  |  |  |  |
| --- | --- | --- | --- | --- | --- | --- | --- | --- | --- | --- |
|  | Odds Ratio | Std. Err. | P>z | [95% Conf. | Interval] | Coef. | Std. Err. | P>z | [95% Conf. | Interval] |
|  |  |  |  |  |  |  |  |  |  |  |
| 1.warf_sensitivity | 0.997 | 0.142 | 0.985 | 0.755 | 1.318 | -0.482 | 0.301 | 0.109 | -1.072 | 0.108 |
| age |  |  |  |  |  |  |  |  |  |  |
| 60-64 years | 0.911 | 0.204 | 0.678 | 0.588 | 1.412 | -0.136 | 0.464 | 0.768 | -1.045 | 0.772 |
| 65-70 years | 0.929 | 0.185 | 0.711 | 0.629 | 1.372 | 0.088 | 0.414 | 0.832 | -0.724 | 0.899 |
| 70-74 years | 0.876 | 0.184 | 0.528 | 0.581 | 1.321 | -0.622 | 0.446 | 0.162 | -1.496 | 0.251 |
| 75+ years | 1.163 | 0.262 | 0.503 | 0.747 | 1.810 | -0.285 | 0.448 | 0.524 | -1.164 | 0.593 |
| ch_index | 1.175 | 0.041 | 0.000 | 1.098 | 1.258 | 0.145 | 0.068 | 0.033 | 0.012 | 0.279 |
| 1.dx1 | 3.133 | 0.834 | 0.000 | 1.859 | 5.280 | 0.280 | 0.444 | 0.528 | -0.590 | 1.149 |
| 1.diab_met | 1.077 | 0.167 | 0.633 | 0.795 | 1.458 | -0.119 | 0.305 | 0.695 | -0.716 | 0.478 |
| 1.malig | 0.779 | 0.119 | 0.102 | 0.578 | 1.051 | 0.265 | 0.315 | 0.400 | -0.353 | 0.883 |

Pseudo R2 0.0438

GOF p-value 0.0452

Log likelihood -3263.482154

AIC 20.58794

BIC -683.1665

**Model for ED CV-Related Costs**

|  | Logit |  |  |  |  | GLM |  |  |  |  |
| --- | --- | --- | --- | --- | --- | --- | --- | --- | --- | --- |
|  | Odds Ratio | Std. Err. | P>z | [95% Conf. | Interval] | Coef. | Std. Err. | P>z | [95% Conf. | Interval] |
|  |  |  |  |  |  |  |  |  |  |  |
| 1.warf_sensitivity | 1.006 | 0.292 | 0.983 | 0.569 | 1.778 | -0.170 | 0.184 | 0.356 | -0.531 | 0.191 |
| age |  |  |  |  |  |  |  |  |  |  |
| 60-64 years | 0.272 | 0.151 | 0.019 | 0.092 | 0.807 | -0.083 | 0.337 | 0.805 | -0.743 | 0.577 |
| 65-70 years | 0.413 | 0.170 | 0.031 | 0.185 | 0.924 | 0.633 | 0.261 | 0.015 | 0.122 | 1.143 |
| 70-74 years | 0.547 | 0.220 | 0.134 | 0.248 | 1.205 | -0.072 | 0.242 | 0.766 | -0.547 | 0.403 |
| 75+ years | 0.996 | 0.385 | 0.991 | 0.467 | 2.123 | -0.179 | 0.244 | 0.462 | -0.657 | 0.298 |
| ch_index | 0.991 | 0.077 | 0.907 | 0.851 | 1.154 | 0.016 | 0.051 | 0.760 | -0.085 | 0.117 |
| 1.dx1 | 0.966 | 0.610 | 0.957 | 0.281 | 3.329 | -0.462 | 0.377 | 0.220 | -1.201 | 0.276 |
| 1.diab_met | 0.983 | 0.323 | 0.959 | 0.517 | 1.871 | 0.140 | 0.222 | 0.528 | -0.295 | 0.576 |
| 1.malig | 0.675 | 0.219 | 0.226 | 0.357 | 1.276 | 0.160 | 0.204 | 0.431 | -0.239 | 0.559 |

Pseudo R2 0.0305

GOF p-value 0.0505

Log likelihood -468.1313348

AIC 16.77654

BIC -172.5488

**Model for Hospital Outpatient CV-Related Costs**

|  | Logit |  |  |  |  | GLM |  |  |  |  |
| --- | --- | --- | --- | --- | --- | --- | --- | --- | --- | --- |
|  | Odds Ratio | Std. Err. | P>z | [95% Conf. | Interval] | Coef. | Std. Err. | P>z | [95% Conf. | Interval] |
|  |  |  |  |  |  |  |  |  |  |  |
| 1.warf_sensitivity | 0.875 | 0.115 | 0.310 | 0.675 | 1.133 | -0.010 | 0.176 | 0.957 | -0.354 | 0.335 |
| age |  |  |  |  |  |  |  |  |  |  |
| 60-64 years | 1.241 | 0.259 | 0.300 | 0.825 | 1.868 | 0.269 | 0.272 | 0.322 | -0.264 | 0.801 |
| 65-70 years | 1.298 | 0.243 | 0.163 | 0.900 | 1.872 | 0.291 | 0.237 | 0.220 | -0.174 | 0.757 |
| 70-74 years | 1.543 | 0.298 | 0.024 | 1.058 | 2.252 | 0.487 | 0.236 | 0.039 | 0.025 | 0.948 |
| 75+ years | 1.547 | 0.330 | 0.041 | 1.018 | 2.351 | 0.183 | 0.260 | 0.483 | -0.328 | 0.693 |
| ch_index | 1.069 | 0.035 | 0.043 | 1.002 | 1.140 | -0.048 | 0.047 | 0.302 | -0.140 | 0.043 |
| 1.dx1 | 1.840 | 0.478 | 0.019 | 1.105 | 3.062 | 0.306 | 0.285 | 0.283 | -0.253 | 0.865 |
| 1.diab_met | 0.976 | 0.141 | 0.865 | 0.735 | 1.296 | -0.237 | 0.183 | 0.196 | -0.596 | 0.122 |
| 1.malig | 0.955 | 0.132 | 0.739 | 0.728 | 1.253 | -0.137 | 0.170 | 0.420 | -0.471 | 0.197 |

Pseudo R2 0.0153

GOF p-value 0.2172

Log likelihood -3618.845613

AIC 18.1897

BIC -1688.6

**Model for Clinic CV-Related Costs**

|  | Logit |  |  |  |  | GLM |  |  |  |  |
| --- | --- | --- | --- | --- | --- | --- | --- | --- | --- | --- |
|  | Odds Ratio | Std. Err. | P>z | [95% Conf. | Interval] | Coef. | Std. Err. | P>z | [95% Conf. | Interval] |
|  |  |  |  |  |  |  |  |  |  |  |
| 1.warf_sensitivity | 1.170 | 0.183 | 0.315 | 0.861 | 1.589 | -0.079 | 0.076 | 0.298 | -0.227 | 0.070 |
| age |  |  |  |  |  |  |  |  |  |  |
| 60-64 years | 0.886 | 0.192 | 0.576 | 0.580 | 1.354 | -0.035 | 0.123 | 0.776 | -0.277 | 0.207 |
| 65-70 years | 1.584 | 0.336 | 0.030 | 1.046 | 2.400 | -0.124 | 0.107 | 0.246 | -0.334 | 0.086 |
| 70-74 years | 1.647 | 0.372 | 0.027 | 1.058 | 2.563 | -0.242 | 0.111 | 0.029 | -0.459 | -0.024 |
| 75+ years | 1.286 | 0.317 | 0.307 | 0.793 | 2.086 | -0.554 | 0.125 | 0.000 | -0.798 | -0.310 |
| ch_index | 1.192 | 0.060 | 0.000 | 1.081 | 1.314 | 0.063 | 0.020 | 0.002 | 0.024 | 0.101 |
| 1.dx1 | 4.047 | 2.445 | 0.021 | 1.238 | 13.224 | 0.195 | 0.145 | 0.177 | -0.088 | 0.479 |
| 1.diab_met | 1.138 | 0.204 | 0.471 | 0.801 | 1.617 | -0.227 | 0.084 | 0.007 | -0.391 | -0.063 |
| 1.malig | 1.049 | 0.173 | 0.771 | 0.759 | 1.450 | -0.029 | 0.079 | 0.714 | -0.183 | 0.126 |

Pseudo R2 0.0459

GOF p-value 0.2006

Log likelihood -6948.546889

AIC 15.65477

BIC -4807.923
