# Supplementary material for: Comparing outcomes and costs among warfarin-sensitive patients versus warfarin-insensitive patients using The Right Drug, Right Dose, Right Time: Using genomic data to individualize treatment (RIGHT) 10K warfarin cohort
Source: PLoS One. 2020 May 19;15(5):e0233316. doi: 10.1371/journal.pone.0233316 (PMC7237006; doi:10.1371/journal.pone.0233316)
Supplement: S3 Table — These tables contain the full regression results (parameter estimates, standard errors, 95% confidence intervals, and p-values for all covariates included in the multivariate models), with models being performed on the empaneled subgroup. A separate table for each regression outcome of interest is included. (DOCX) [file pone.0233316.s005.docx]

**S3 Table. Complete regression results for the empaneled subgroup**

**Model for Experience a Major Bleeding Event**

|  | Odds Ratio | Std. Err. | P>z | [95% Conf. | Interval] |
| --- | --- | --- | --- | --- | --- |
|  |  |  |  |  |  |
| 1.warf_sensitivity | 0.938 | 0.284 | 0.834 | 0.518 | 1.699 |
| age |  |  |  |  |  |
| 60-64 years | 1.103 | 0.614 | 0.860 | 0.370 | 3.286 |
| 65-70 years | 0.835 | 0.438 | 0.731 | 0.299 | 2.332 |
| 70-74 years | 1.341 | 0.650 | 0.545 | 0.518 | 3.470 |
| 75+ years | 2.285 | 1.072 | 0.078 | 0.911 | 5.732 |
| ch_index | 1.131 | 0.070 | 0.045 | 1.002 | 1.277 |
| 1.dx1 | 1.380 | 0.658 | 0.500 | 0.542 | 3.513 |
| 1.diab_met | 1.257 | 0.386 | 0.457 | 0.689 | 2.293 |
| 1.malig | 0.935 | 0.293 | 0.830 | 0.506 | 1.727 |

**Model for Number of Major Bleeding Events (entire study sample)**

|  | IRR | Std. Err. | P>z | [95% Conf. | Interval] |
| --- | --- | --- | --- | --- | --- |
|  |  |  |  |  |  |
| 1.warf_sensitivity | 1.226 | 0.528 | 0.637 | 0.527 | 2.852 |
| age |  |  |  |  |  |
| 60-64 years | 1.258 | 0.944 | 0.760 | 0.289 | 5.479 |
| 65-70 years | 1.510 | 1.014 | 0.539 | 0.405 | 5.632 |
| 70-74 years | 2.177 | 1.371 | 0.217 | 0.633 | 7.482 |
| 75+ years | 2.817 | 1.834 | 0.112 | 0.786 | 10.091 |
| ch_index | 1.177 | 0.125 | 0.126 | 0.955 | 1.449 |
| 1.dx1 | 3.790 | 2.912 | 0.083 | 0.841 | 17.088 |
| 1.diab_met | 1.070 | 0.463 | 0.876 | 0.458 | 2.499 |
| 1.malig | 1.667 | 0.752 | 0.257 | 0.688 | 4.037 |

Pseudo R2 0.0268

**Model for Number of Major Bleeding Events (for those who experienced a bleed)**

|  | IRR | Std. Err. | P>z | [95% Conf. | Interval] |
| --- | --- | --- | --- | --- | --- |
|  |  |  |  |  |  |
| 1.warf_sensitivity | 1.135 | 0.214 | 0.503 | 0.784 | 1.643 |
| age |  |  |  |  |  |
| 60-64 years | 1.295 | 0.538 | 0.534 | 0.573 | 2.924 |
| 65-70 years | 1.927 | 0.705 | 0.073 | 0.941 | 3.948 |
| 70-74 years | 1.704 | 0.556 | 0.102 | 0.899 | 3.230 |
| 75+ years | 1.256 | 0.405 | 0.480 | 0.667 | 2.363 |
| ch_index | 1.069 | 0.033 | 0.033 | 1.005 | 1.137 |
| 1.dx1 | 1.860 | 0.471 | 0.014 | 1.132 | 3.057 |
| 1.diab_met | 0.870 | 0.168 | 0.472 | 0.595 | 1.271 |
| 1.malig | 1.887 | 0.360 | 0.001 | 1.298 | 2.742 |

Pseudo R2 0.1073

**Model for Total All-Cause Costs**

|  | Coef. | Std. Err. | P>z | [95% Conf. | Interval] |
| --- | --- | --- | --- | --- | --- |
|  |  |  |  |  |  |
| 1.warf_sensitivity | -0.014 | 0.107 | 0.896 | -0.224 | 0.196 |
| age |  |  |  |  |  |
| 60-64 years | -0.055 | 0.180 | 0.759 | -0.407 | 0.297 |
| 65-70 years | -0.102 | 0.161 | 0.526 | -0.418 | 0.214 |
| 70-74 years | -0.122 | 0.162 | 0.452 | -0.440 | 0.196 |
| 75+ years | 0.117 | 0.170 | 0.490 | -0.216 | 0.451 |
| ch_index | 0.091 | 0.028 | 0.001 | 0.036 | 0.146 |
| 1.dx1 | 0.453 | 0.210 | 0.031 | 0.042 | 0.865 |
| 1.diab_met | -0.117 | 0.112 | 0.295 | -0.337 | 0.102 |
| 1.malig | 0.065 | 0.112 | 0.565 | -0.156 | 0.285 |

Log likelihood -6077.221841

AIC 21.39621

BIC -2749.139

**Model for Inpatient All-Cause Costs**

|  | Logit |  |  |  |  | GLM |  |  |  |  |
| --- | --- | --- | --- | --- | --- | --- | --- | --- | --- | --- |
|  | Odds Ratio | Std. Err. | P>z | [95% Conf. | Interval] | Coef. | Std. Err. | P>z | [95% Conf. | Interval] |
|  |  |  |  |  |  |  |  |  |  |  |
| 1.warf_sensitivity | 1.201 | 0.228 | 0.337 | 0.827 | 1.743 | -0.055 | 0.142 | 0.698 | -0.333 | 0.223 |
| age |  |  |  |  |  |  |  |  |  |  |
| 60-64 years | 0.834 | 0.261 | 0.563 | 0.452 | 1.541 | 0.092 | 0.238 | 0.699 | -0.375 | 0.559 |
| 65-70 years | 0.701 | 0.198 | 0.208 | 0.403 | 1.218 | 0.110 | 0.214 | 0.607 | -0.309 | 0.528 |
| 70-74 years | 0.778 | 0.223 | 0.380 | 0.444 | 1.363 | 0.018 | 0.214 | 0.932 | -0.402 | 0.438 |
| 75+ years | 0.811 | 0.245 | 0.487 | 0.448 | 1.466 | 0.300 | 0.226 | 0.184 | -0.142 | 0.743 |
| ch_index | 1.100 | 0.056 | 0.062 | 0.995 | 1.216 | 0.030 | 0.035 | 0.390 | -0.038 | 0.098 |
| 1.dx1 | 1.990 | 0.873 | 0.117 | 0.842 | 4.702 | 0.464 | 0.256 | 0.070 | -0.038 | 0.965 |
| 1.diab_met | 1.143 | 0.229 | 0.505 | 0.771 | 1.694 | -0.078 | 0.150 | 0.602 | -0.373 | 0.216 |
| 1.malig | 0.840 | 0.165 | 0.374 | 0.572 | 1.233 | 0.135 | 0.152 | 0.374 | -0.162 | 0.432 |

Pseudo R2 0.0173

GOF p-value 0.4875

Log likelihood -4067.720936

AIC 21.18297

BIC -1326.262

**Model for ED All-Cause Costs**

| Logit |  |  |  |  | GLM |  |  |  |  |
| --- | --- | --- | --- | --- | --- | --- | --- | --- | --- |
| Odds Ratio | Std. Err. | P>z | [95% Conf. | Interval] | Coef. | Std. Err. | P>z | [95% Conf. | Interval] |
|  |  |  |  |  |  |  |  |  |  |
| 0.989 | 0.227 | 0.960 | 0.630 | 1.550 | -0.081 | 0.181 | 0.653 | -0.436 | 0.273 |
|  |  |  |  |  |  |  |  |  |  |
| 0.491 | 0.183 | 0.057 | 0.236 | 1.021 | -0.654 | 0.296 | 0.027 | -1.235 | -0.073 |
| 0.472 | 0.155 | 0.022 | 0.248 | 0.899 | 0.051 | 0.254 | 0.842 | -0.448 | 0.549 |
| 0.510 | 0.167 | 0.040 | 0.268 | 0.970 | -0.477 | 0.269 | 0.076 | -1.004 | 0.051 |
| 0.438 | 0.156 | 0.021 | 0.218 | 0.881 | -0.406 | 0.284 | 0.153 | -0.962 | 0.150 |
| 1.054 | 0.058 | 0.339 | 0.946 | 1.175 | 0.061 | 0.047 | 0.195 | -0.031 | 0.154 |
| 2.072 | 0.793 | 0.057 | 0.979 | 4.386 | -0.392 | 0.277 | 0.157 | -0.935 | 0.151 |
| 1.265 | 0.301 | 0.323 | 0.794 | 2.015 | -0.089 | 0.184 | 0.629 | -0.449 | 0.271 |
| 0.983 | 0.237 | 0.942 | 0.612 | 1.578 | 0.041 | 0.186 | 0.825 | -0.324 | 0.406 |

Pseudo R2 0.0241

GOF p-value 0.1568

Log likelihood -856.7336378

AIC 16.82978

BIC -368.3276

**Model for Hospital Outpatient All-Cause Costs**

|  | Logit |  |  |  |  | GLM |  |  |  |  |
| --- | --- | --- | --- | --- | --- | --- | --- | --- | --- | --- |
|  | Odds Ratio | Std. Err. | P>z | [95% Conf. | Interval] | Coef. | Std. Err. | P>z | [95% Conf. | Interval] |
|  |  |  |  |  |  |  |  |  |  |  |
| 1.warf_sensitivity | 1.002 | 0.187 | 0.992 | 0.695 | 1.443 | 0.069 | 0.166 | 0.679 | -0.257 | 0.395 |
| age |  |  |  |  |  |  |  |  |  |  |
| 60-64 years | 1.099 | 0.326 | 0.751 | 0.615 | 1.964 | -0.307 | 0.287 | 0.285 | -0.869 | 0.256 |
| 65-70 years | 1.208 | 0.326 | 0.484 | 0.711 | 2.052 | -0.408 | 0.251 | 0.105 | -0.900 | 0.085 |
| 70-74 years | 1.329 | 0.366 | 0.301 | 0.775 | 2.280 | -0.170 | 0.249 | 0.493 | -0.658 | 0.317 |
| 75+ years | 1.488 | 0.437 | 0.176 | 0.837 | 2.645 | 0.036 | 0.255 | 0.887 | -0.464 | 0.536 |
| ch_index | 1.272 | 0.076 | 0.000 | 1.132 | 1.429 | 0.047 | 0.042 | 0.273 | -0.037 | 0.130 |
| 1.dx1 | 1.551 | 0.658 | 0.301 | 0.675 | 3.564 | 0.400 | 0.286 | 0.162 | -0.160 | 0.960 |
| 1.diab_met | 0.913 | 0.181 | 0.647 | 0.619 | 1.347 | -0.438 | 0.169 | 0.009 | -0.768 | -0.107 |
| 1.malig | 0.980 | 0.191 | 0.919 | 0.669 | 1.436 | -0.072 | 0.163 | 0.659 | -0.393 | 0.248 |

Pseudo R2 0.0436

GOF p-value 0.3757

Log likelihood -3401.472417

AIC 19.05851

BIC -1518.051

**Model for Clinic All-Cause Costs**

|  | Coef. | Std. Err. | P>z | [95% Conf. | Interval] |
| --- | --- | --- | --- | --- | --- |
|  |  |  |  |  |  |
| 1.warf_sensitivity | -0.026 | 0.076 | 0.731 | -0.175 | 0.123 |
| age |  |  |  |  |  |
| 60-64 years | -0.092 | 0.126 | 0.463 | -0.339 | 0.154 |
| 65-70 years | -0.256 | 0.113 | 0.024 | -0.478 | -0.033 |
| 70-74 years | -0.347 | 0.114 | 0.002 | -0.571 | -0.123 |
| 75+ years | -0.365 | 0.119 | 0.002 | -0.599 | -0.131 |
| ch_index | 0.136 | 0.019 | 0.000 | 0.098 | 0.175 |
| 1.dx1 | -0.183 | 0.147 | 0.213 | -0.470 | 0.105 |
| 1.diab_met | -0.070 | 0.078 | 0.371 | -0.223 | 0.083 |
| 1.malig | 0.120 | 0.079 | 0.130 | -0.035 | 0.276 |

Log likelihood -5124.98871

AIC 18.04917

BIC -3089.475

**Model for Total CV-Related Costs**

|  | Logit |  |  |  |  | GLM |  |  |  |  |
| --- | --- | --- | --- | --- | --- | --- | --- | --- | --- | --- |
|  | Odds Ratio | Std. Err. | P>z | [95% Conf. | Interval] | Coef. | Std. Err. | P>z | [95% Conf. | Interval] |
|  |  |  |  |  |  |  |  |  |  |  |
| 1.warf_sensitivity | 0.855 | 0.204 | 0.512 | 0.535 | 1.366 | -0.439 | 0.219 | 0.045 | -0.869 | -0.009 |
| age |  |  |  |  |  |  |  |  |  |  |
| 60-64 years | 0.924 | 0.307 | 0.813 | 0.482 | 1.773 | 0.069 | 0.380 | 0.855 | -0.675 | 0.814 |
| 65-70 years | 1.689 | 0.557 | 0.112 | 0.884 | 3.224 | 0.335 | 0.334 | 0.316 | -0.319 | 0.988 |
| 70-74 years | 2.690 | 1.001 | 0.008 | 1.297 | 5.577 | -0.064 | 0.325 | 0.845 | -0.701 | 0.574 |
| 75+ years | 2.150 | 0.814 | 0.043 | 1.023 | 4.517 | 0.078 | 0.356 | 0.827 | -0.620 | 0.775 |
| ch_index | 1.385 | 0.130 | 0.001 | 1.151 | 1.666 | 0.061 | 0.054 | 0.256 | -0.044 | 0.167 |
| 1.dx1 | 1.000 | (empty) |  |  |  | 0.931 | 0.389 | 0.017 | 0.168 | 1.693 |
| 1.diab_met | 1.010 | 0.268 | 0.970 | 0.601 | 1.699 | -0.280 | 0.223 | 0.209 | -0.717 | 0.157 |
| 1.malig | 0.790 | 0.198 | 0.348 | 0.484 | 1.292 | 0.063 | 0.232 | 0.787 | -0.392 | 0.517 |

Pseudo R2 0.0669

GOF p-value 0.6939

Log likelihood -4522.694829

AIC 19.32919

BIC -1388.798

**Model for Inpatient CV-Related Costs**

|  | Logit |  |  |  |  | GLM |  |  |  |  |
| --- | --- | --- | --- | --- | --- | --- | --- | --- | --- | --- |
|  | Odds Ratio | Std. Err. | P>z | [95% Conf. | Interval] | Coef. | Std. Err. | P>z | [95% Conf. | Interval] |
|  |  |  |  |  |  |  |  |  |  |  |
| 1.warf_sensitivity | 0.979 | 0.196 | 0.917 | 0.661 | 1.450 | -0.617 | 0.330 | 0.062 | -1.264 | 0.030 |
| age |  |  |  |  |  |  |  |  |  |  |
| 60-64 years | 1.058 | 0.362 | 0.868 | 0.541 | 2.070 | -0.064 | 0.588 | 0.914 | -1.215 | 1.088 |
| 65-70 years | 1.197 | 0.365 | 0.556 | 0.658 | 2.177 | 0.269 | 0.508 | 0.597 | -0.728 | 1.265 |
| 70-74 years | 1.126 | 0.348 | 0.701 | 0.614 | 2.064 | -0.326 | 0.516 | 0.528 | -1.338 | 0.686 |
| 75+ years | 1.257 | 0.403 | 0.476 | 0.670 | 2.356 | 0.067 | 0.550 | 0.903 | -1.011 | 1.145 |
| ch_index | 1.208 | 0.058 | 0.000 | 1.099 | 1.327 | 0.001 | 0.069 | 0.994 | -0.135 | 0.136 |
| 1.dx1 | 3.855 | 1.385 | 0.000 | 1.907 | 7.795 | 0.785 | 0.473 | 0.097 | -0.141 | 1.711 |
| 1.diab_met | 0.923 | 0.194 | 0.704 | 0.611 | 1.394 | -0.160 | 0.320 | 0.618 | -0.787 | 0.467 |
| 1.malig | 0.679 | 0.146 | 0.072 | 0.445 | 1.035 | 0.357 | 0.370 | 0.335 | -0.369 | 1.082 |

Pseudo R2 0.0626

GOF p-value 0.3805

Log likelihood -1660.864226

AIC 20.37639

BIC -246.0623

**Model for ED CV-Related Costs**

|  | Logit |  |  |  |  | GLM |  |  |  |  |
| --- | --- | --- | --- | --- | --- | --- | --- | --- | --- | --- |
|  | Odds Ratio | Std. Err. | P>z | [95% Conf. | Interval] | Coef. | Std. Err. | P>z | [95% Conf. | Interval] |
|  |  |  |  |  |  |  |  |  |  |  |
| 1.warf_sensitivity | 0.927 | 0.348 | 0.841 | 0.444 | 1.936 | -0.328 | 0.245 | 0.181 | -0.809 | 0.153 |
| age |  |  |  |  |  |  |  |  |  |  |
| 60-64 years | 0.243 | 0.158 | 0.030 | 0.068 | 0.872 | -0.227 | 0.387 | 0.557 | -0.985 | 0.531 |
| 65-70 years | 0.338 | 0.172 | 0.033 | 0.125 | 0.917 | 0.676 | 0.352 | 0.055 | -0.014 | 1.367 |
| 70-74 years | 0.352 | 0.181 | 0.042 | 0.129 | 0.962 | -0.470 | 0.303 | 0.120 | -1.063 | 0.123 |
| 75+ years | 0.359 | 0.197 | 0.062 | 0.122 | 1.054 | -0.269 | 0.371 | 0.468 | -0.996 | 0.458 |
| ch_index | 0.995 | 0.103 | 0.958 | 0.811 | 1.219 | 0.033 | 0.066 | 0.623 | -0.097 | 0.162 |
| 1.dx1 | 0.485 | 0.510 | 0.492 | 0.062 | 3.810 | -0.942 | 0.660 | 0.154 | -2.237 | 0.352 |
| 1.diab_met | 0.835 | 0.342 | 0.660 | 0.375 | 1.863 | 0.009 | 0.258 | 0.972 | -0.497 | 0.515 |
| 1.malig | 0.666 | 0.278 | 0.330 | 0.294 | 1.509 | 0.260 | 0.257 | 0.311 | -0.243 | 0.763 |

Pseudo R2 0.0493

GOF p-value 0.2125

Log likelihood -291.1844989

AIC 17.21054

BIC -77.41297

**Model for Hospital Outpatient CV-Related Costs**

|  | Logit |  |  |  |  | GLM |  |  |  |  |
| --- | --- | --- | --- | --- | --- | --- | --- | --- | --- | --- |
|  | Odds Ratio | Std. Err. | P>z | [95% Conf. | Interval] | Coef. | Std. Err. | P>z | [95% Conf. | Interval] |
|  |  |  |  |  |  |  |  |  |  |  |
| 1.warf_sensitivity | 0.844 | 0.155 | 0.356 | 0.590 | 1.210 | -0.056 | 0.239 | 0.814 | -0.525 | 0.412 |
| age |  |  |  |  |  |  |  |  |  |  |
| 60-64 years | 1.297 | 0.407 | 0.407 | 0.702 | 2.397 | 0.014 | 0.417 | 0.973 | -0.803 | 0.832 |
| 65-70 years | 1.572 | 0.443 | 0.109 | 0.904 | 2.732 | 0.193 | 0.369 | 0.601 | -0.530 | 0.915 |
| 70-74 years | 1.863 | 0.525 | 0.027 | 1.072 | 3.237 | 0.390 | 0.356 | 0.273 | -0.308 | 1.089 |
| 75+ years | 1.901 | 0.560 | 0.029 | 1.067 | 3.388 | -0.068 | 0.375 | 0.855 | -0.803 | 0.666 |
| ch_index | 1.030 | 0.046 | 0.515 | 0.943 | 1.125 | -0.037 | 0.066 | 0.582 | -0.166 | 0.093 |
| 1.dx1 | 2.343 | 0.814 | 0.014 | 1.185 | 4.631 | 0.387 | 0.358 | 0.279 | -0.314 | 1.088 |
| 1.diab_met | 0.878 | 0.169 | 0.499 | 0.602 | 1.280 | -0.401 | 0.243 | 0.099 | -0.878 | 0.075 |
| 1.malig | 1.001 | 0.190 | 0.996 | 0.690 | 1.453 | -0.083 | 0.233 | 0.722 | -0.539 | 0.373 |

Pseudo R2 0.0228

GOF p-value 0.684

Log likelihood -1972.265898

AIC 18.26973

BIC -764.5279

**Model for Clinic CV-Related Costs**

|  | Logit |  |  |  |  | GLM |  |  |  |  |
| --- | --- | --- | --- | --- | --- | --- | --- | --- | --- | --- |
|  | Odds Ratio | Std. Err. | P>z | [95% Conf. | Interval] | Coef. | Std. Err. | P>z | [95% Conf. | Interval] |
|  |  |  |  |  |  |  |  |  |  |  |
| 1.warf_sensitivity | 1.018 | 0.237 | 0.940 | 0.645 | 1.605 | -0.072 | 0.097 | 0.461 | -0.263 | 0.119 |
| age |  |  |  |  |  |  |  |  |  |  |
| 60-64 years | 0.929 | 0.302 | 0.820 | 0.490 | 1.758 | 0.058 | 0.171 | 0.736 | -0.277 | 0.392 |
| 65-70 years | 1.749 | 0.570 | 0.086 | 0.924 | 3.312 | -0.010 | 0.149 | 0.947 | -0.302 | 0.282 |
| 70-74 years | 2.680 | 0.968 | 0.006 | 1.320 | 5.440 | -0.150 | 0.148 | 0.310 | -0.441 | 0.140 |
| 75+ years | 1.663 | 0.582 | 0.146 | 0.837 | 3.301 | -0.497 | 0.157 | 0.002 | -0.805 | -0.188 |
| ch_index | 1.219 | 0.095 | 0.012 | 1.045 | 1.421 | 0.045 | 0.025 | 0.068 | -0.003 | 0.093 |
| 1.dx1 | 5.632 | 5.813 | 0.094 | 0.745 | 42.586 | 0.221 | 0.175 | 0.205 | -0.121 | 0.563 |
| 1.diab_met | 1.194 | 0.306 | 0.488 | 0.723 | 1.973 | -0.272 | 0.101 | 0.007 | -0.469 | -0.075 |
| 1.malig | 0.888 | 0.215 | 0.622 | 0.552 | 1.427 | -0.082 | 0.101 | 0.414 | -0.280 | 0.115 |

Pseudo R2 0.0661

GOF p-value 0.019

Log likelihood -3588.616907

AIC 15.57843

BIC -2223.343
